# Multi-omics profiling reveals key signaling pathways in ovarian cancer controlled by STAT3

**DOI:** 10.7150/thno.33444

**Published:** 2019-07-28

**Authors:** Tiangong Lu, Armand Bankhead, Mats Ljungman, Nouri Neamati

**Affiliations:** 1 Department of Medicinal Chemistry, College of Pharmacy, Rogel Cancer Center, University of Michigan, Ann Arbor, MI 48109-2800, USA; 2 Department of Computational Medicine and Bioinformatics, University of Michigan, Ann Arbor, MI 48109-2800, USA; 3 Department of Biostatistics, School of Public Health, University of Michigan, Ann Arbor, MI 48109-2800, USA; 4 Departments of Radiation Oncology, Rogel Cancer Center, University of Michigan Medical School and Department of Environmental Health Sciences, School of Public Health, University of Michigan, Ann Arbor, MI 48109-2800, USA

**Keywords:** STAT3, Ovarian cancer, CRISPR-Cas9, Multi-omic genome-wide analysis, *STAT3* knockout

## Abstract

Inhibiting STAT3 signaling reduces tumor progression, metastasis and chemoresistance, however the precise molecular mechanism has not been fully delineated in ovarian cancer.

Methods: In this study, we generated *STAT3* knockout (KO) ovarian cancer cell lines. Effects of *STAT3* KO on cell proliferation, migration and spheroid formation were assessed *in vitro* and effects on *in vivo* tumor growth were tested using several tumor xenograft models. We used multi-omic genome-wide profiling to identify multi-level (Bru-Seq, RNA-Seq, and MS Proteomic) expression signatures of *STAT3* KO ovarian cancer cells.

Results: We observed that deletion of *STAT3* blocked cell proliferation and migration *in vitro* and suppressed tumor growth in mice. Deletion of *STAT3* transcriptionally suppressed key genes involved in EMT, cell cycle progression, E2F signaling, and altered stemness markers. Notably, KO of *STAT3* resulted in modulation of the expression of other STAT family members.

Conclusion: Our study presents a rich, multi-faceted summary of the molecular mechanisms impacted by *STAT3* deletion and provides new insight for *STAT3*'s potential as a therapeutic target in ovarian cancer.

## Introduction

Ovarian cancer is the fifth leading cause of cancer death in women and the most lethal gynecological malignancy in the United States 1, 2. Approximately 90% of ovarian cancers are epithelial ovarian carcinomas (EOCs) 3, 4. About 70% of EOC cases are diagnosed at an advanced stage with metastasis to adjacent organs or the abdominal cavity through peritoneal fluid 3. First-line treatment with platinum and taxane initially improves therapeutic outcomes, but often results in drug resistance that leads to relapse 5. In recurrent cancer, treatments such as anti-angiogenic agents, poly(ADP-ribose) polymerase inhibitors, and immunological therapies show limited efficacy 4. Thus, there is an urgent need to identify novel targets and develop more effective therapeutics to improve patient outcomes.

Signal transducer and activator of transcription 3 (STAT3) participates in a wide variety of physiological processes 6-8. A notable feature of STAT3 is that it not only transduces cytoplasmic signals from extracellular stimuli, but also functions as a latent transcription factor regulating gene expression 9, 10. Early embryonic lethality of *STAT3* knockout (KO) transgenic mice suggests an essential role in development 11. Constitutively activated STAT3 mediates oncogenic transformation in mice 12. It has been shown that STAT3 plays a critical role in promoting tumor proliferation, survival, inflammatory response, immunity, cancer stem cells, angiogenesis, and invasion in many human malignancies 9, 13. STAT3 is expressed and constitutively activated in EOC cell lines compared to normal ovarian surface epithelial cells, and induces downstream mediator expression, including Bcl-xL and cyclin D1 14. Importantly, phosphorylation activated STAT3 (p-STAT3) positively correlates with disease aggressiveness and negatively correlates with survival in ovarian cancer patients 15, 16. Higher levels of STAT3 and p-STAT3 observed in patients' metastatic tumors versus primary tumors suggest a critical role of STAT3 in ovarian tumor progression/metastasis 17. Abrogation of STAT3 expression/activity using siRNA, shRNA, or small molecules inhibits EOC cell migration and invasion *in vitro* and decreases tumor growth *in vivo* 15, 17, 18. Moreover, PI3K/AKT, BRAF, SRC, and MEK/ERK-targeted therapies can induce a “feedback activation” of STAT3 contributing to drug-resistance and promoting cell survival 19, 20. Previous studies have shed light on the role of STAT3 dysregulation in cancer progression and evaluated it as a target for potential therapeutic interventions using genetic manipulation (Supplemental Table S1). Although various aspects of STAT3 signaling have been reported in several cancer models, the precise molecular mechanisms and consequences of inhibiting STAT3 are not fully understood in EOC.

Herein, using CRISPR-Cas9 genome editing, we generated multiple *STAT3* KO ovarian cancer cell lines. Our results demonstrate that deletion of *STAT3* prevents ovarian cancer cell proliferation, migration and spheroid formation *in vitro*, and blocks tumor growth *in vivo.* Using RNA-Seq profiling, liquid chromatography-mass spectrometry proteomic profiling, and bromouridine-based nascent RNA sequencing (Bru-Seq) 21, 22 we have characterized the transcriptional and translational response to *STAT3* KO in EOCs. Deletion of *STAT3* alters the transcription of other STAT family member genes, transcriptionally suppresses genes involved in epithelial-mesenchymal transition (EMT), cell cycle progression and E2F signaling. Furthermore, *STAT3* KO alters expression of stemness markers (ALDH1A and CD44). Altogether, our genome-wide, multi-omic analysis reveals a signature of *STAT3* regulatory programs and uncovers new signaling networks of STAT3 promoting ovarian tumor growth, progression, and metastasis. Our study provides a rich, multi-faceted summary of the molecular mechanisms impacted by STAT3 inhibition and will further guide the evaluation of STAT3 as a therapeutic target in ovarian cancer.

## Results

### Generation of STAT3 KO ovarian cancer cell lines

To elucidate the functional role of STAT3 in ovarian cancer, CRISPR-Cas9 induced genome-editing was used to knock out *STAT3* in the ovarian cancer cell lines HEY, OVCAR3, OVCAR8 and SKOV3. To avoid potential off-target effects, three guide RNA sequences were designed to target three different exons in *STAT3* DNA. Cells were co-transfected with *STAT3*-targeting guide RNAs, Cas9 nuclease mRNA, and orange fluorescent protein (OFP) cleavage selection vectors. OFP-positive cells having Cas9 cleavage were detected and enriched by fluorescence-activated cell sorting (FACS) (Supplemental Figure S1A and S1B). As shown in Supplemental Figure S1A, SKOV3 cells modified with gRNA1 and Cas9 expressed the lowest amount of STAT3, with a 13.5-fold lower expression than wildtype (WT). A total of four ovarian cancer *STAT3* KO single cell clones were generated from HEY, OVCAR3, OVCAR8 and SKOV3 (Supplemental Figure S1C).

### STAT3 deletion reduces cell proliferation, migration and spheroid formation in vitro

**Figure 1:**
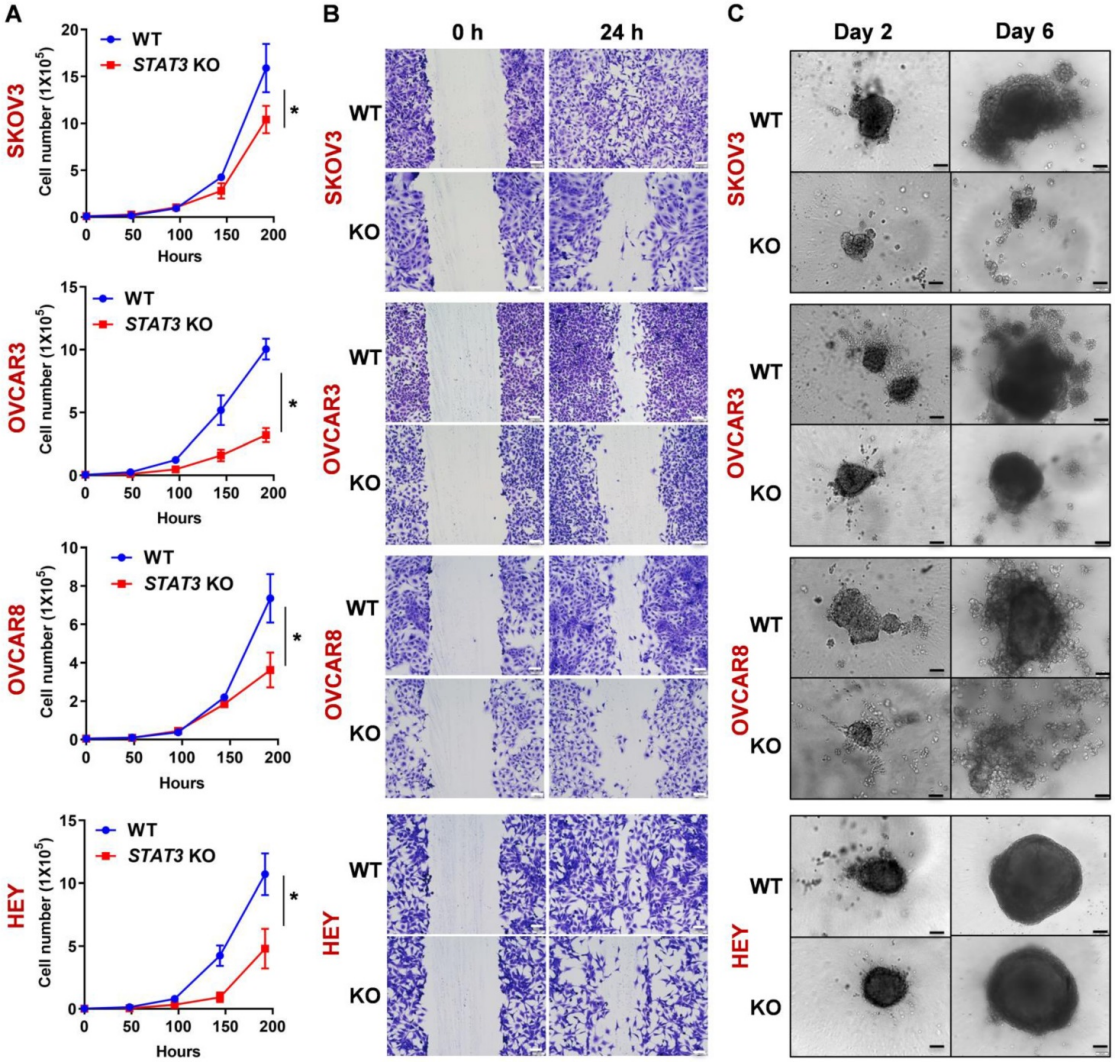
** Deletion of STAT3 reduces cell proliferation, migration, and spheroid formation of ovarian cancer cells in vitro. (A).** The proliferation rate of SKOV3, OVCAR3, OVCAR8 and HEY WT/ *STAT3* KO cells was evaluated by a cell proliferation assay. The graph displays the cell numbers versus time in h. **(B).** Migration capability of WT and *STAT3* KO cells was determined in a wound-healing assay. The panels on the left show the wound at 0 h after the scratch and the right panels show the wound after 24 h. Scale bar, 100 μm. A bar diagram with statistical analysis is provided in Supplemental Figure S2A. **(C).** Spheroid formation capability of WT and *STAT3* KO cells was determined in a 3D spheroid assay. Spheroid growth was imaged at Days 2, 4 and 6. Scale bar, 100 μm. Luminescence representing cell viability of the same experiment was measured using the CellTiter-Glo® 3D Cell Viability Assay and is presented with statistical analysis in Supplemental Figure S2B.

To assess the effect of *STAT3* KO on proliferation rates, we compared doubling times of the WT and KO cells. SKOV3 *STAT3* KO cells had a prolonged doubling time (29.5 h) compared to WT cells (26.7 h) within the tested period (192 h) (Figure 1A). A similar trend was observed in all four cell lines (Figure 1A). Since STAT3 has been shown to be necessary for migration and invasion 23, we confirmed that KO of *STAT3* altered the migratory ability of ovarian cancer cell lines in an *in vitro* wound-healing assay. Deletion of *STAT3* prevented wound healing, further supporting the role of STAT3 in cell growth and migration (Figure 1B, Supplemental Figure S2A). Importantly, STAT3 is required for ovarian cancer cells to grow in 3D. Spheroid formation capability was significantly inhibited in *STAT3* KO cell lines in a 3D culture system. (Figure 1C, Supplemental Figure S2B). OVCAR3, OVCAR8 and SKOV3 WT cells were able to form large and compact spheroids after two days, while the spheroids from corresponding KO cells remained small, loosely associated aggregates. Though no difference in size was observed between HEY WT and *STAT3* KO spheroids (Figure 1C), the cell viability (Figure S2B) of *STAT3* KO spheroids was significantly reduced.

### STAT3 KO inhibits tumor growth in mouse xenograft models

**Figure 2:**
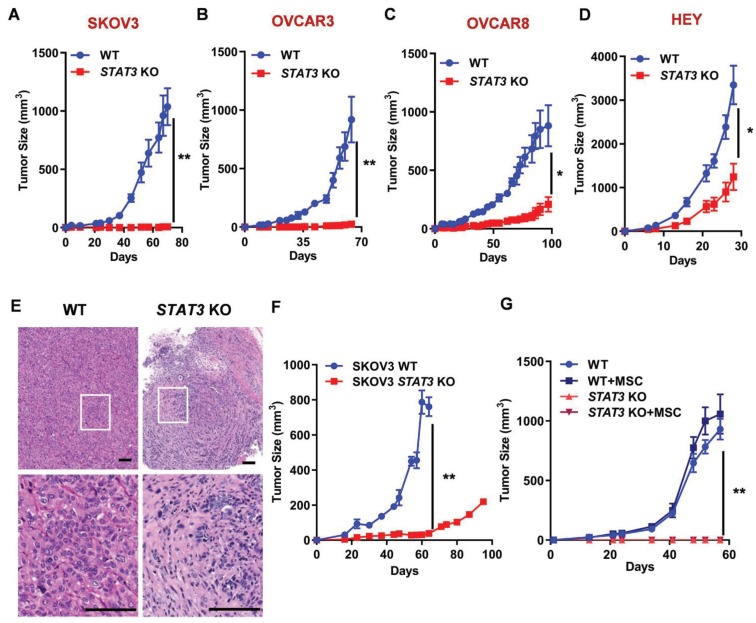
** STAT3 KO causes tumor growth inhibition in mouse xenograft models of ovarian cancer. (A-D).** Growth curves of tumors in nude mice (n = 5) injected with SKOV3 (A), OVCAR3 (B), OVCAR8 (C) and HEY (D) WT/ *STAT3* KO cells. **(E)** H&E staining of SKOV3 WT/ *STAT3* KO xenograft tumor tissue sections. The bottom panels show a higher magnification of the boxed area in the upper panel. Overall histologic tumor size and cellular density of the *STAT3* KO specimen was markedly reduced in comparison to the WT specimen histologically. The overall appearance of the *STAT3* KO specimen was that of collapse, with fewer tumor cells and more stroma than that of the WT specimen. Cellular features between the two specimens were also observably different. Relatively less anisocytosis and anisokaryosis, with smaller cells, many of which contained more dense cytoplasm and fewer abnormal nuclear features were observed in *STAT3* KO specimen. Scale bar, 100 μm. **(F)** Growth curves of tumors in huNOG mice injected with SKOV3 WT/ *STAT3* KO cells (n = 2). **(G)** NSG mice (n = 5) were co-injected with SKOV3 WT/ *STAT3* KO cells, with or without ovarian cancer patient-derived mesenchymal stem cells (MSC). Statistical significance was calculated using Student's t-test. Error bars indicate mean ± SEM (standard error of mean). *p < 0.05, **p < 0.01.

Since *STAT3* KO inhibits spheroid formation capability, we investigated its effect on tumor growth in xenograft models in either NOG or athymic nude mice. *STAT3* KO significantly reduced tumor growth in all ovarian cancer cell-derived xenografts compared to WT control (Figure 2A-D, Supplemental Figure S3). The biggest difference between WT and *STAT3* KO was observed in SKOV3 and OVCAR3 xenografts: the tumor did not grow after KO of *STAT3*. Immunohistochemical staining of SKOV3 tumor tissues revealed significant histologic differences between the WT and *STAT3* KO tumor specimens (Figure 2E, a full pathology report is provided in Supplemental data 1).* STAT3* KO tumors were composed of fewer tumor cells within a more abundant collagenous stroma, while WT tumors presented with abundant tumor cells with vacuolated cytoplasm within a scant fine fibrovascular stroma. These findings suggest that STAT3 expression is positively correlated with tumor growth *in vivo*.

Cancer progression relies on tumor-intrinsic effects (e.g. genetic aberrations) and the tumor microenvironment (e.g. cell-cell interaction and immune response) 24. Several lines of evidence suggests that inhibition of STAT3 triggers activation of dendritic cells, T cells, natural killer cells, enhancing tumor immune suppression, therefore, inhibiting tumor growth and metastasis 25. To analyze the immunological consequences of *STAT3* deletion in EOCs, SKOV3 WT and *STAT3* KO cells were implanted into humanized immune NOG mice engrafted with human CD34+ hematopoietic stem cells, which were used to reconstitute the human tumor immune microenvironment *in vivo*. After 60 days, *STAT3* KO xenografts were significantly suppressed compared to control tumors expressing *STAT3* (Figure 2F). Mesenchymal stem cells (MSCs) are important stromal facilitators of tumor development. In response to multiple signals produced by cancer cells (e.g. growth factors, chemokines and cytokines), multipotent progenitor cells are recruited and enhance tumor growth and metastatic progression 26. To test the hypothesis that STAT3-mediated cytokine regulation may affect tumor growth, SKOV3 WT and *STAT3* KO cells were co-cultured with MSCs and implanted into NSG mice. In the presence of MSC, parental SKOV3 cells exhibited a modest increase in tumor growth (Figure 2G). No growth of *STAT3* KO tumors were observed despite the presence of MSC. These observations indicate that deletion of *STAT3* in the tumor makes cells unresponsive to the growth stimulatory effect of MSC and support the hypothesis that STAT3 plays a critical role in the growth of tumors regardless of microenvironment signaling.

### Multi-omic Profiling of STAT3 KO in SKOV3 cells

**Figure 3:**
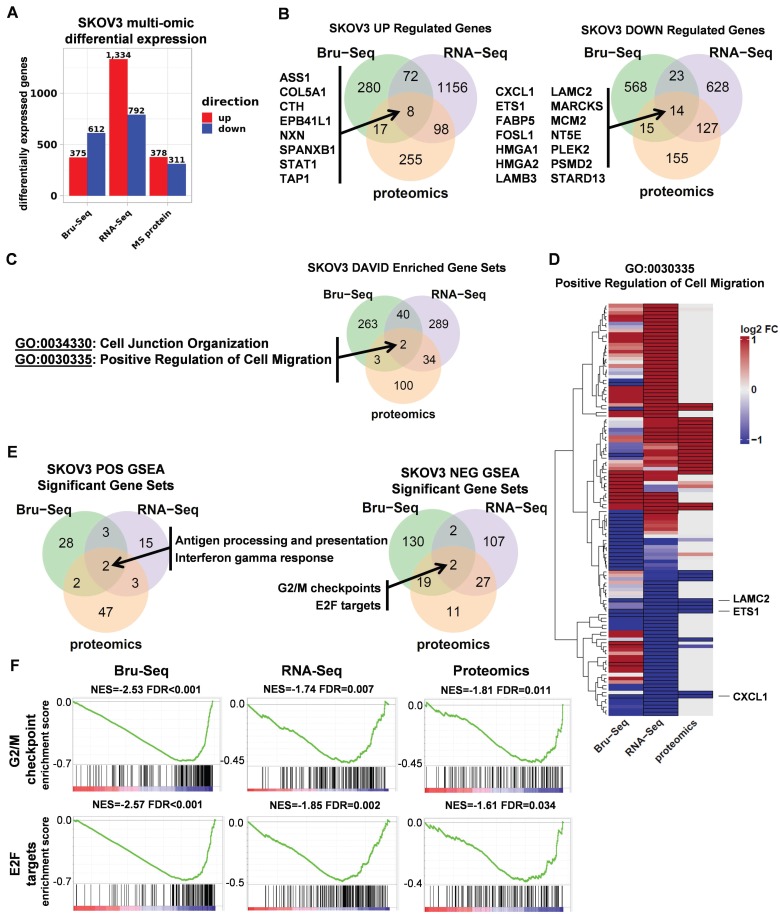
** RNA and protein expression profiles of SKOV3 STAT3 parental and KO cells. (A).** Differential expression of genes as a result of *STAT3* KO across different platforms. **(B).** A total of 22 genes were commonly up (8 genes) or down (14 genes) regulated across all three profiling platforms. **(C).** Using DAVID, two Gene Ontology categories “Cell Junction Organization” and “Positive Response to Cell Migration” were commonly enriched for genes differentially expressed in all 3 platforms. **(D)** Heatmap showing log2 fold changes of 116 genes belonging to the Positive Regulation of Cell Migration gene set that were differentially expressed in at least one platform. **(E)** Overlap of GSEA enriched gene sets for SKOV3 *STAT3* KO. Up-regulated genes included KEGG Antigen Processing and Presentation, and hallmark Interferon Gamma Response. Down-regulated genes included 2 hallmark gene sets: G2/M Checkpoint, and E2F Targets. **(F)** GSEA enrichment plots for each enriched gene set and platform. NES = normalized enrichment score; FDR= false discovery rate. Differential expression thresholds used per platform are described in methods.

To elucidate the functional role of STAT3 in regulating gene expression in ovarian cancer, we profiled SKOV3 *STAT3* KO and WT cells using nascent RNA Bru-Seq (*N* =1), RNA-Seq (*N* =3) and MS proteomics (*N* =3). The numbers of differentially expressed genes varied greatly across all three platforms. Although Bru-Seq and RNA-Seq identified thousands of significantly changing genes (987 and 2 126 respectively) and MS proteomics identified 689 differentially expressed proteins, a total of 22 genes and gene products were identified as significantly changing across all three data types (Figure 3A-B, Supplemental Table S2).

For each platform, over-representation analysis (ORA) was performed using DAVID on combined up and down-regulated gene lists using Gene Ontology Biological Process and KEGG gene sets. Consistent with *STAT3*'s known involvement in cell migration, the Cell Junction Organization and Positive Regulation of Cell Migration categories were commonly enriched across all 3 platforms (Figure 3C, Supplemental Table S3). Figure 3D illustrates fold changes of 116 genes in the GO:0030335 gene set with differential expression observed in at least one platform. Though differences in coverage, fold change intensity, and even fold change direction across all 3 data types were observed, there was overall agreement between platforms for the majority of genes in the major up and down-regulated gene dendrogram clusters (Supplemental Table S4). This overall concordance between platforms reinforces the functional relevance of *STAT3* in cell migration and is consistent with findings in the wound healing assays described above. *ETS1, LAMC2,* and *CXCL1* are the most significantly differentially expressed across all three platforms.

Gene Set Enrichment Analysis (GSEA) was used to identify STAT3 direction-specific regulatory programs in common across all 3 platforms. Two gene sets, “KEGG antigen processing and presentation” and “Hallmark interferon gamma response,” were identified as upregulated after deletion of *STAT3* (Figure 3E, Supplemental Figure S4A). Cancer development often impairs function of antigen-presenting cells, such as dendritic cells (DC), on recognizing, processing and presenting antigen, therefore disrupting protective immune responses. Activation of STAT3 suppresses DC maturation and promotes DC dysfunction 27. Interferon-γ is involved in tumor control by enhancing cellular immune response against transformed cells. STAT1 and STAT3 are competitively activated by Interferon-γ 28, 29. Significantly up-regulated genes in both of these gene sets observed across all three profiling platforms indicates *STAT3* KO cells significantly differed from WT cells in initiating adaptive immune responses. Two common hallmark gene sets were significantly (FDR adjusted p-value < 0.05) down-regulated in response to *STAT3* KO: “G2/M Checkpoint” and “E2F Targets,” (Figure 3E-F, Supplemental Table S5). We also observed that the Epithelial Mesenchymal Transition gene set trended toward reduced expression across platforms but did not survive multiple testing correction (Supplemental Table S5). Cell cycle progression and EMT promote cancer cell proliferation and metastasis. Decreased synthesis and translation of cell cycle and EMT genes suggests a potential explanation to the observation that *STAT3* KO cells were less aggressive and invasive than their parental cells both *in vitro* and *in vivo*.

### Transcriptional response to aberrant STAT3 expression in ovarian cancer cells

**Figure 4:**
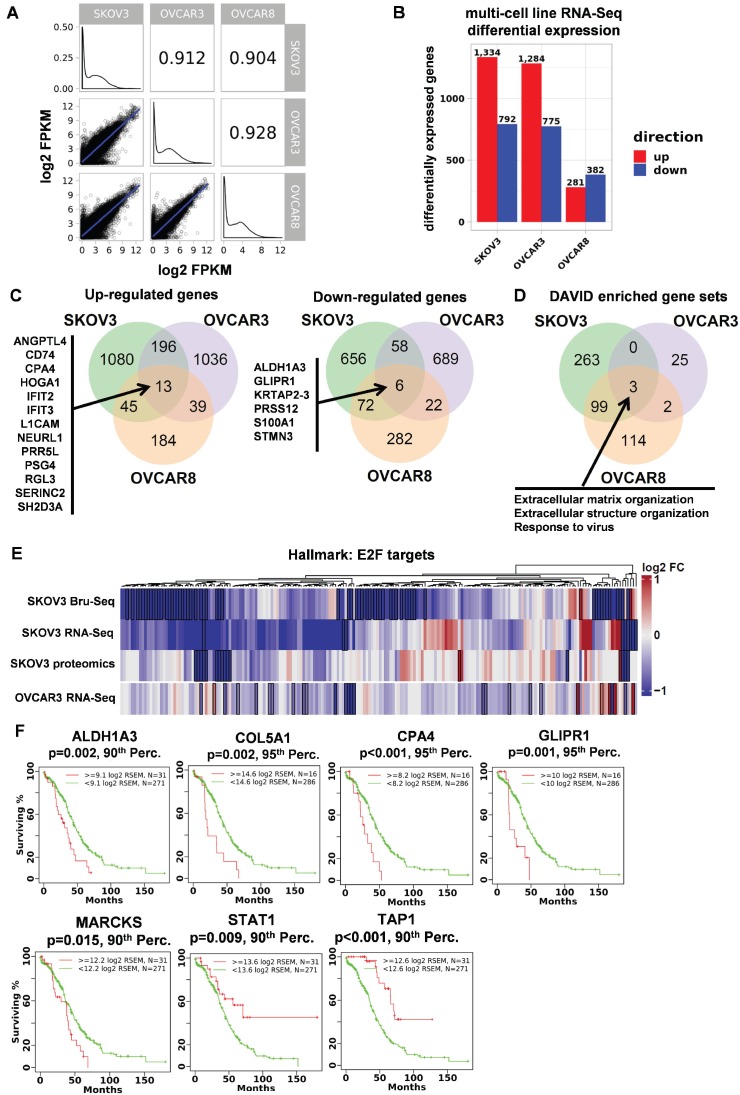
** Common genes and pathways altered across three ovarian cancer cell lines in response to STAT3 deletion. (A)** Transcriptional profile comparison between parental ovarian cell lines shows a high degree of similarity. **(B)** *STAT3* KO RNA-Seq differential expression across three ovarian cancer cell lines. **(C)** 19 differentially expressed genes were observed across all 3 cell lines in response to *STAT3* KO. **(D)** Using DAVID, differentially expressed genes were commonly enriched in three Gene Ontology categories. FDR-adjusted *p*-values for “GO:0030198 Extracellular Matrix Organization” in SKOV3, OVCAR3 and OVCAR8 are 1.51 × 10^-8^, 0.0597 and 8.3 × 10^-6^, respectively. FDR-adjusted *p*-values for “GO:0043062 Extracellular Structure Organization” in SKOV3, OVCAR3 and OVCAR8 are 1.72 × 10^-8^, 0.0634 and 4.9 × 10^-8^, respectively. FDR-adjusted *p*-values for “GO:0009615 Response to Virus” in SKOV3, OVCAR3 and OVCAR8 are 0.0245, 0.0930, and 0.0363, respectively. **(E)** The E2F Targets hallmark gene set was significantly enriched for down-regulated genes using GSEA (FDR adjusted *p*-value < 0.1) in SKOV3 and OVCAR3 cell lines. **(F)** Kaplan-Meier survival plots of 7 genes have significant associations with patient survival analyzed from TCGA.

Next, we characterized the transcription signature of *STAT3* in multiple ovarian cancer cells. Given SKOV3, OVCAR3 and OVCAR8 cell lines were observed with more active response towards deletion of *STAT3* both *in vitro* and *in vivo*, the effects on gene expression to *STAT3* KO of these 3 cell lines were examined using RNA-Seq profiling. Global steady state RNA profiles between all 3 parental cell lines show a high degree of similarity by Spearman correlation (ρ = 0.904 to ρ = 0.928) (Figure 4A). However, the effects on gene expression of *STAT3* KO varied greatly in both the magnitude of differential expression and the genes impacted: 663 genes changed significantly in OVCAR8, whereas at least 3 times as many genes changed significantly for SKOV3 and OVCAR3 (2 126 and 2 059, respectively) (Figure 4B). Figure 4C shows 19 differentially expressed genes in common across all 3 cell lines. ORA DAVID revealed 3 common gene sets (Figure 4D, Supplemental Table S3), 2 of which were related to the extracellular matrix reinforcing STAT3's functional relevance to cell adhesion and migration in ovarian cancer. “Response to virus” was also commonly enriched, indicating that *STAT3* is associated with virus-induced complications. Using GSEA, the hallmark gene set “E2F Targets” was enriched (q-value < 0.01) in both SKOV3 KO and OVCAR3 KO, but not in OVCAR8 KO cells (Figure 4E, Supplemental Figure S4B, Table S5). It is clear that most genes regulated by E2F are downregulated in the *STAT3* KO cell lines indicating a link between STAT3 and E2F target expression (Figure 4E). We noted that OVCAR8 had the lowest levels of STAT3 expression (11.4 mean FPKM of parental cells) compared to SKOV3 and OVCAR3 (41.6, 15.1 FPKM, respectively) and hypothesize that STAT3 plays a less prominent role in OVCAR8's transcriptional program.

### Multi-omic analyses identified 41 genes differentially expressed in response to STAT3 KO and 7 significantly associated with patient survival

Across all three data types and three cell lines, a total of 41 genes and gene products were identified as significantly changing in response to *STAT3* KO (Figure 3B and 4C, Supplemental Table S6). Most of their functional relation to *STAT3* has not been previously described in the literature. The most up-regulated genes/proteins in SKOV3 *STAT3* KO cells observed across platforms was TAP1 (Figure 3B). TAP1 (transporter associated with antigen processing 1) is an immune surveillance protein and key components of antigen processing. Two pairs of family member genes, HMGA1/2 and LAMB3/C2, were found to be downregulated at both RNA and protein levels in SKOV3 *STAT3* deleted cells (Figure 3B). High-mobility group A proteins (HMGA) are key factors in early embryogenesis, and overexpression of HMGA proteins is consistently observed in all malignant tumors and tightly correlates with metastatic progression 30. *STAT3* is a critical downstream target of *HMGA1* and their expression is positively correlated in leukemia patient samples, while *STAT3* was reported to co-localize with *HMGA2* 31, 32. *LAMB3* and *LAMC2* encode the β3 and γ2 chains of laminin-5, which is a major adhesive component of basement membrane 33. Abnormal expression and interaction of laminin-5 with integrins α3β1 and α6β4 promote cancer development 34.

13 genes were up-regulated across all three cell lines (Figure 4C). Recent studies confirmed a positive correlation between L1 cell adhesion molecule (L1CAM) and STAT3: inhibiting L1CAM by monoclonal antibody *in vivo* reduced p-STAT3, while over-expression of L1CAM activated the STAT3/NF-κB signaling pathway 35, 36. *STAT3* and *ANGPTL4* (encode Angiopoietin-like 4) are directly correlated in glioblastoma patients and increased expression of these genes results in poor survival 37. Among the 6 commonly downregulated genes in three cell lines, *ALDH1A3* has been shown to be involved in *STAT3* signaling (Figure 4C). Activated STAT3 is more highly expressed in ALDH+ cells over ALDH- cells, and inhibition of STAT3 by small molecule inhibitor, Stattic, reduced ALDH1A3 expression in lung cancer stem cells 38.

7 of the 41 genes have significant associations with ovarian cancer patient survival analyzed from TCGA, and their Kaplan-Meier survival plots are shown in Figure 4F. While the expression of ALDH1A3, COL5A1, CPA4, GLIPR1, HABP4, MARCKS and PYGB are unfavorably associated with patient survival, TAP1 and STAT1 expression significantly associated with improved overall survival. Furthermore, patient data from ICGC and Tothill were downloaded to validate relevance to *in vivo* cohorts 39. ALDH1A3, MARCKS, and COL5A1 were associated with reduced survival across all 3 datasets underscoring their relevance to ovarian cancer disease. TAP1 was associated with increased patient survival across all 3 datasets. (Supplemental Figure S5) 41 differentially expressed genes were processed using the ClueGO v2.5.3 cytoscape analysis plugin to generate a concise visual representation of gene set enrichment (Supplemental Figure S6). Graph nodes represent gene sets enriched for STAT3 regulated genes and edges represent overlap between genes sets. ClueGO identified twelve statistically significant and functionally distinct clusters including oncogene-induced cell senescence, metabolism, cell differentiation, and immune response.

### Deletion of STAT3 suppresses the epithelial-mesenchymal transition

**Figure 5:**
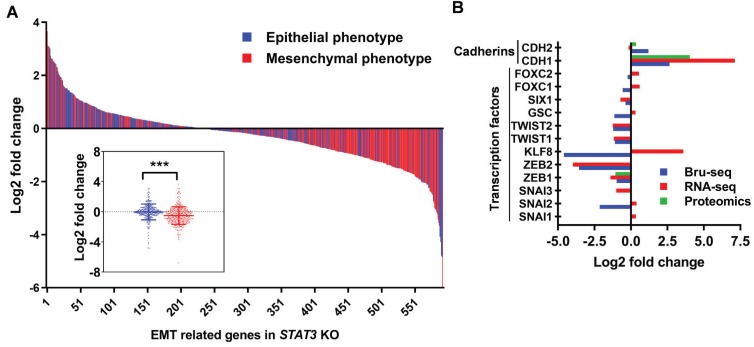
** STAT3 KO suppresses epithelial-mesenchymal transition. (A).** Log2 fold change profile of nascent RNA encoding EMT-regulated genes in SKOV3 *STAT3* KO samples. Genes colored red were annotated as pertaining to a mesenchymal phenotype (n=311), while genes colored blue were annotated as pertaining to an epithelial phenotype (n=284) in cancer cells. 14 epithelial- or mesenchymal-related annotated gene sets obtained from the Molecular Signatures Database (MSigDB, GSEA - Broad Institute) were used to generate a list of 747 genes and corresponding epithelial/mesynchemal annotations. Gene list is provided in Supplemental Table S7. Boxplot at bottom left represents log2 fold change average of genes regulating epithelial and mesenchymal phenotypes in SKOV3 *STAT3* KO/WT. *** indicate a *p*-value < 0.001, unpaired Student's t-test, two-tailed *p*-value. **(B).** EMT markers and EMT-TFs governing EMT progression are downregulated in SKOV3 *STAT3* knockout cells at the RNA and protein levels.

As described above, the gene set “epithelial to mesenchymal transition” was downregulated at the transcriptional and protein levels in *STAT3* KO cells (Supplemental Figure S7A). We annotated 747 genes as pertaining to an epithelial or a mesenchymal phenotype using 14 gene sets from the Molecular Signatures Database (MSigDB, GSEA - Broad Institute) (Supplemental Table S7). Nascent RNA expression from 595 genes (165 were significantly differentially expressed) was detected by SKOV3 Bru-Seq. These genes are presented as a waterfall plot ranked in order of log2 fold change and colored by epithelial/mesenchymal phenotype (Figure 5A). We observe a trend of genes pertaining to the mesenchymal phenotype having significantly reduced expression compared to the genes that promote an epithelial phenotype in response to *STAT3* KO (*p*-value ≤ 0.0005). RNA-Seq analysis confirmed that the expression of mesenchymal genes were significantly suppressed in OVCAR3 (*p*-value = 0.005) and SKOV3 (*p*-value = 0.05), but not in OVCAR8 (not significant, *p*-value = 0.2) *STAT3* KO cells (Supplemental Figure S7B). EMT is controlled by a network of EMT-inducing transcription factors (EMT-TFs) that regulate the expression of proteins involved in cell-cell contact, cytoskeleton structure, and extracellular matrix degradation 40. Loss of E-cadherin and gain of N-cadherin (cadherin switching) is considered a marker of EMT progression. Though we observed a slightly elevated nascent RNA and protein level of CDH2 (N-cadherin, mesenchymal marker), the epithelial marker, CDH1 (E-cadherin), was significantly upregulated in *STAT3* KO cells (Figure 5B). Repression of several reported primary EMT-TFs 40 was observed upon *STAT3* deletion as illustrated in Figure 5B: downregulation of *SNAI2&3*, *ZEB1&2, KLF8, TWIST1&2, GSC, SIX1,* and *FOXC1* was observed at RNA or/and nascent RNA level, while decreased ZEB1 protein expression was also detected. Collectively, our data suggests that loss of *STAT3* inhibits EMT progression by suppressing the transcription of mesenchymal phenotype genes and EMT-TFs.

### Deletion of STAT3 suppresses cell cycle progression in ovarian cancer

**Figure 6:**
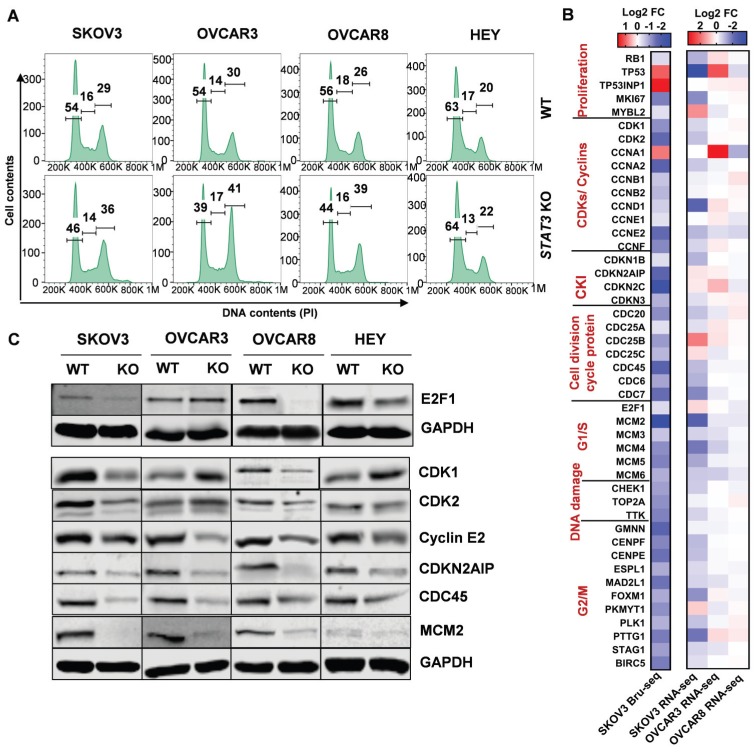
** STAT3 KO downregulates expression of genes involved in the G2/M phase. (A).** Cell cycle profiling revealed G2/M enrichment in *STAT3* KO cells. Cells were fixed, stained with propidium iodide, and DNA content was analysed by flow cytometry. Statistical analysis is provided in Supplemental Table S8. **(B)** Key cell cycle mediators coding genes were mostly down regulated in SKOV3 *STAT3* KO cells compared to WT cells. Colors represented log2 fold change differences between *STAT3* KO cell lines and parental cell lines. The impact of *STAT3* KO varied greatly in the magnitude of differential expression between Bru-seq and RNA-seq, two scale bars are used. **(C).** Key cell cycle mediators were suppressed in *STAT3* KO cells. Protein expression levels were determined by Western blot.

Using GSEA, the “G2/M checkpoint” hallmark gene set was significantly suppressed by *STAT3* deletion in both SKOV3 and OVCAR3 cells (*q*-values 0.01, 0.05, respectively) (Supplemental Figure S8A, Supplemental Table S5) suggesting that *STAT3* promotes the transcription of genes involved in cell cycle progression. Furthermore, *STAT3* KO arrest cells in the G2/M phase as compared to the respective WT cells (Figure 6A, Supplemental Table S8). The largest increase in G2/M phase was observed for OVCAR3 *STAT3* KO cells, with 41% in the G2/M population in *STAT3* KO cells compared to 30% in the WT samples.

These findings prompted us to investigate the transcriptional profile of cell cycle genes. Consistent with a cell cycle arrest phenotype, there was relatively low gene expression of CDKs (CDK1 and CDK2), most cyclins (cyclins A1, A2, B1, B2, D, E1, E2 and F), cell division cycle (CDC) proteins and checkpoint proteins upon *STAT3* KO (Figure 6B). Nascent transcription of cyclin-dependent kinase inhibitor (CDKI) genes were also inhibited. Next, we examined the expression of key cell cycle mediators regulating different phases in *STAT3* KO cells (Figure 6C, Supplemental Figure S8B). Reduced expression of E2F1 was observed in all *STAT3* KO cells except OVCAR3. Protein expression of CDK1 and 2 was not significantly changed. Cyclin E2, a G1/S cyclin that binds CDK2, was downregulated in all *STAT3* KO cells. CKIs are well characterized for their role as negative regulators through the cell cycle. We found that the nascent transcriptional expression of several CKIs, CDKN2AIP (p16-interacting protein), CDKN3 and p27^Kip1^ (encoded by *CDKN1B*), were dramatically reduced in all *STAT3* KO cells. No p27^Kip1^ expression were detected in SKOV3 cells (Supplemental Figure S8B). CDC proteins, CDC25c and CDC45, were decreased upon *STAT3* KO. Additionally, the expression of several mini-chromosome maintenance (MCM) family proteins, critical regulators of DNA replication and cell division, was suppressed in SKOV3 *STAT3* KO cells. Especially, a significantly decreased expression of MCM2 were observed in SKOV3, OVCAR3 and OVCAR8 *STAT3* KO cells. Survivin (encoded by *BIRC5*), a direct downstream target of oncogenic STAT3, was downregulated in all *STAT3* KO cells (Supplemental Figure S8B). Taken together, our data suggests that deletion of *STAT3* suppresses cell cycle progression in ovarian cancer.

### Deletion of STAT3 alters the expression of other STAT family members

**Figure 7:**
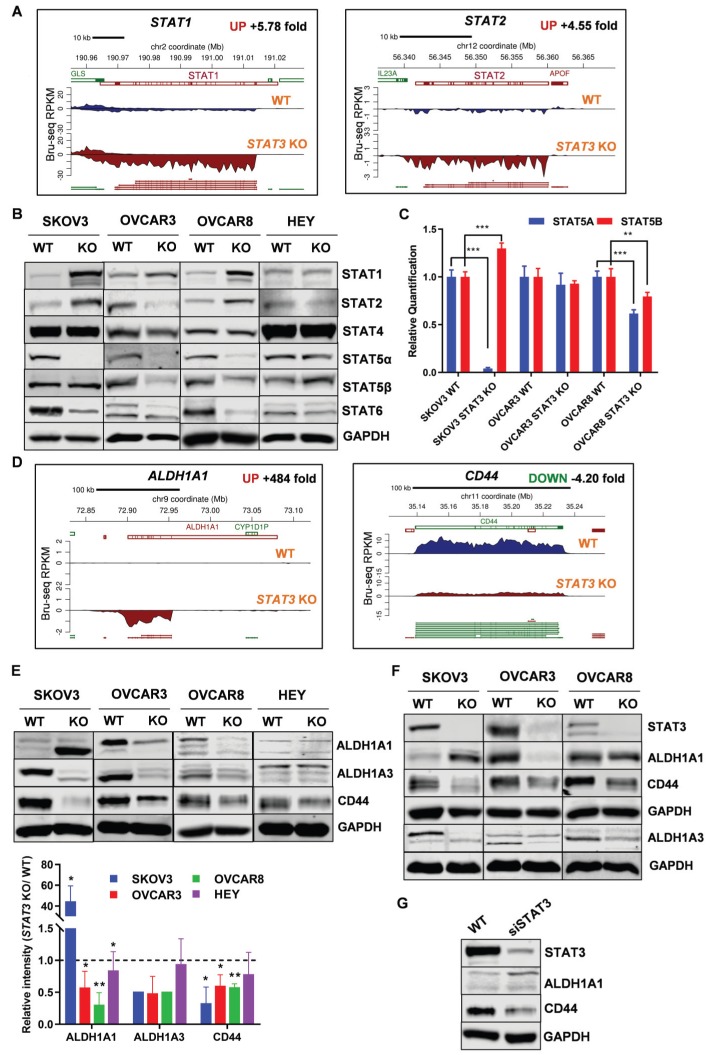
** STAT3 KO alters expression of STAT family members and stemness-like markers. ( A).** *STAT1* and *STAT2* nascent RNA are upregulated in SKOV3 *STAT3* KO cells as determined by Bru-Seq analysis. **(B).** Changes in protein expression of STAT family members in WT/ *STAT3* KO cells. **(C).** RT-PCR of *STAT5A* and *STAT5B* expression in *STAT3* KO cells compared to control cells. **(D).** *ALDH1A1* nascent RNA is upregulated and *CD44* is downregulated in SKOV3 *STAT3* KO cells. **(E).** Protein expression of ALDH1A1, ALDH1A3 and CD44 in WT/ *STAT3* KO cells. **(F).** Expression of STAT3, ALDH1A1 and CD44 in nude xenograft tumors from ovarian cancer cells with *STAT3* deletion. **(G).** Protein expression of STAT3, ALDH1A1 and CD44 upon *STAT3* knock-down*.* SKOV3 cells were treated with 30 nM STAT3 siRNA for 72 h and lysed for Western blot. Gene map is from RefSeq Genes (UCSC genome browser, http://genome.ucsc.edu/) and RPKM directionality indicates strand. RPKM=reads per kilobase of transcript per million mapped reads.

We evaluated the transcription and expression of STAT family members in *STAT3* KO cells (Supplemental Figure S8C). Bru-Seq analysis of SKOV3 *STAT3* KO cells revealed that *STAT1* and *STAT2* were among the top 50 significantly up-regulated genes compared to WT cells, with a fold change of 5.8 and 4.6, respectively (Figure 7A). STAT1 RNA and protein expression also increased 10.6 and 2.2-fold in SKOV3 *STAT3* KO, respectively, as confirmed by RNA-Seq and proteomics analysis (Supplemental Figure S8C). The protein levels of STAT1 were higher in all KO cell lines except for HEY (Figure 7B). STAT2 levels were higher in SKOV3 and OVCAR8 *STAT3* KO cells, lower in OVCAR3, and did not change in HEY cells. STAT4 levels were not affected by *STAT3* KO; however, STAT6 expression decreased (Figure 7B). Notably, the transcription of *STAT5A* was significantly downregulated in SKOV3 *STAT3* KO cells, with a fold change of 5.4 and 17.7, respectively (Supplemental Figure S8C). Decreased STAT5α expression was also observed in all *STAT3* KO cells with the exception of HEY (Figure 7B). To rule out that the dramatic decrease of *STAT5A* in *STAT3* KO cells was due to the off-target effect of CRISPR-Cas9 genome editing, the RNA transcript levels of *STAT5A* and its highly conserved gene *STAT5B* was evaluated by RT-PCR. A downward trend was observed for the transcription of *STAT5A* across all tested *STAT3* KO cells, though the decrease in OVCAR3 *STAT3* KO cells was not significant (Figure 7C). No discernable trend or correlation on the transcription of *STAT5B* was observed with *STAT3* KO. Although we noted a significantly increased expression of *STAT5B* in SKOV3 cells, the *STAT5B* expression was decreased in OVCAR3 and OVCAR8 *STAT3* KO cells (Figure 7C). Taken together, our findings indicate that deletion of *STAT3* alters the expression of STAT family members.

### STAT3 KO alters the stem-like properties of ovarian cancer cells

Aldehyde dehydrogenase 1 (ALDH1) family is considered as a marker for cancer stem cells (CSC). Bru-Seq profiling of SKOV3 *STAT3* KO cells revealed that *ALDH1A1* was the second most upregulated gene with a 484-fold change (Figure 7D). We confirmed that ALDH1A1 protein expression in SKOV3 *STAT3* KO cells increased both *in vitro* and *in vivo* (Figure 7E-F). However, *STAT3* loss in OVCAR3 and OVCAR8 resulted in reduced ALDH1A1 protein levels in both cells and xenograft tumors, while the ALDH1A1 protein expression in HEY cells was low and did not change (Figure 7E-F). To rule out that the up-regulation observed in SKOV3 *STAT3* KO cells was due to single clone behavior, we knocked down *STAT3* with siRNA in SKOV3 cells. Suppression of *STAT3* with siRNA also increased ALDH1A1 expression (Figure 7G). Since ALDH1A3 was identified among the top downregulated genes, we tested its protein expression. Reduced ALDH1A3 protein expression was observed in all *STAT3* KO cell lines except HEY (Figure 7E). Decreased expression of ALDH1A3 were further observed in *STAT3* KO cell-derived tumors (Figure 7F).

CD44 is recognized as a typical CSC surface marker in various cancers. We found that *CD44* is among the top 100 downregulated genes (4.2-fold in Bru-seq and 3.19-fold in RNA-seq) and protein (2.2-fold) after *STAT3* deletion in SKOV3 cells (Figure 7D and Supplemental Table S2). In addition, protein expression of CD44 from *STAT3* KO cells and xenograft tumors were decreased in all tested *STAT3* KO cells, with SKOV3 *STAT3* KO expressing the lowest levels of CD44 (Figure 7E-F). Furthermore, *STAT3* siRNA knockdown reduced CD44 protein expression in SKOV3 cells (Figure 7G). Collectively, our findings indicate that loss of *STAT3* in ovarian cancer cells alters stem like properties. This includes reducing ALDH1A3 and CD44 expression, however, modulation of ALDH1A1 is cell line dependent. The precise interactions among STAT3, ALDH1A3, and CD44 in ovarian cancer require further assessment.

### TCGA clinical analysis confirmed an unified set of STAT3-related regulatory programs in four cancer types

**Figure 8:**
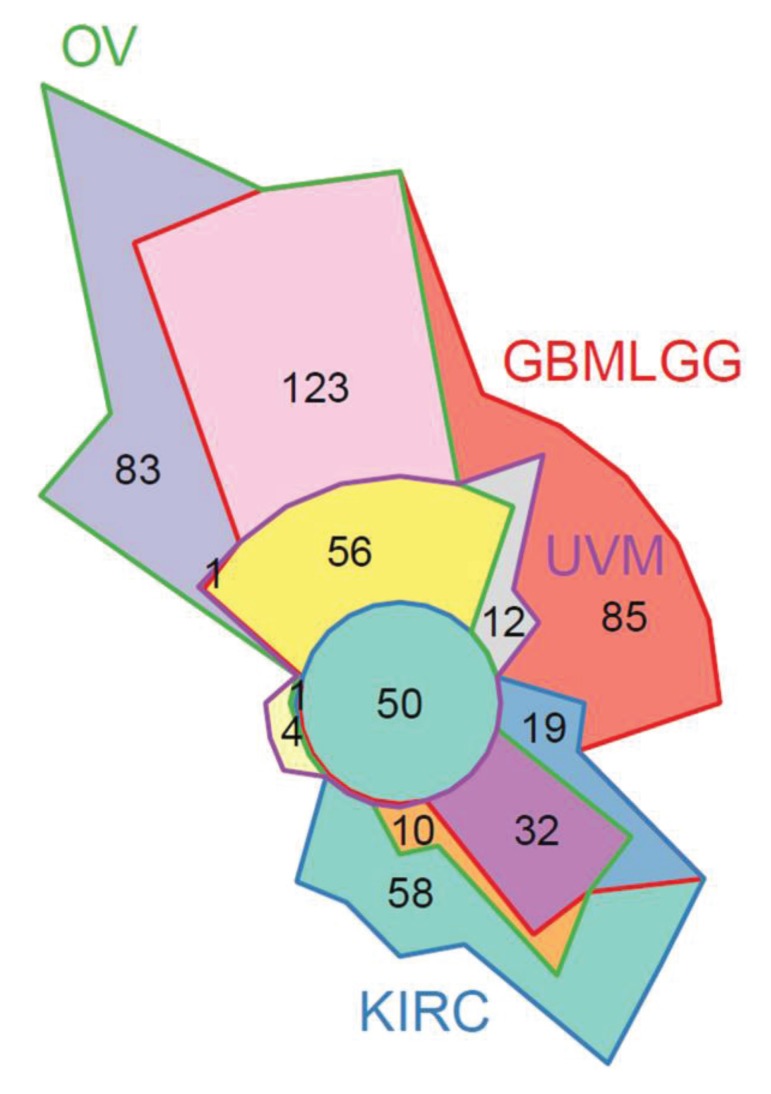
** 50 significant STAT3 co-expression gene sets in common between 4 diseases.** STAT3 co-expression modelled using Gene Set Enrichment Analysis (GSEA) to identify gene sets enriched for genes correlated with STAT3 expression in ovarian (OV), glioblastoma and lower grade glioma (GBMLGG), uveal melanoma (UVM), and kidney renal clear cell carcinoma (KIRC) TCGA cohorts. A full list of gene sets is provided in Supplemental Figure S9B.

To further validate our *in vitro* findings, we evaluated STAT3 RNA-Seq co-expression in patient gene expression samples. The Cancer Genome Atlas (TCGA) patient clinical and gene expression data were downloaded from the Genome Data Analysis Center (GDAC) Firehose 41. Significant associations with high STAT3 expression and reduced survival were identified in the merged Glioma (GBMLGG), Uveal Melanoma (UVM), and Kidney renal clear cell carcinoma (KIRC) cohorts (Supplemental Figure S9A). A *STAT3* co-expression analysis was performed in ovarian (OV), GBMLGG, UVM, and KIRC TCGA cohorts using RNA-Seq expression data by quantifying the enrichment of genes correlated with STAT3 expression in MSigDB gene sets. GSEAv2.2.3 was used with v6 gene sets sourced from MSigDB to quantify enrichment significance using 10 000 gene set permutations were performed with weighted mode scoring and Pearson metric. Fifty gene sets were identified in common between all four cancer types indicating a unified set of STAT3-related regulatory programs (Figure 8, Supplemental Figure S9B). As expected, gene sets pertaining to STAT-related and JAK/STAT signaling were enriched including: GO:0097696 STAT Cascade, Hallmark IL6/JAK/STAT3 Signaling, KEGG JAK/STAT Signaling Pathway. Pathways relating to cellular immunity also observed in SKOV3, OVCAR3, and OVCAR8 *STAT3* KO cells were enriched in four cancer types: interferon gamma response, immune response, immune cell activation and differentiation. In agreement with our findings on suppressed EMT by *STAT3* deletion, pathways and gene sets pertaining to cell adhesion and migration were also significantly enriched (Supplemental Figure S9B).

## Discussion

In this study, a combination of CRISPR-Cas9-mediated gene KO and multi-omic genome-wide profiling enabled us to identify multi-level (Bru-Seq, RNA-Seq, and MS Proteomic) expression signatures of parental and *STAT3* KO ovarian cancer cells. Our data demonstrate that STAT3 expression is tightly associated with tumor proliferation and aggressiveness in both i*n vitro* and *in vivo* ovarian cancer models. We confirmed multiple previously identified STAT3-associated genes and, more importantly, identified new gene sets and 41 genes differentially regulated in response to *STAT3* KO involved in a wide range of cellular processes.

In this study, Cas9-gRNA complexes were delivered into cultured human cells via lipid-mediated transfection of Cas9 mRNA/gRNA complexes and fluorophore cleavage selection vectors. Single cell selection was further performed on positive fluorescent cells. Cas9 delivery as mRNA were reported with increased genomic cleavage efficiency and reduced off-target cleavage rate due to the observed faster protein depletion when compared with DNA plasmid transfection or lentiviral delivery 42. Single-cell-derived population stability provides high accuracy in genomic analysis. Average responses from subpopulations with mixed KOs and wild types may not be as informative and consistent. However, the variations from cell to cell may result single clonal behavior, which should be considered when evaluating the general applicability of subsequent experimental results. This can be overcome by comparing genotype and phenotype of several clones or between multiple cell lines.

We observed suppressed cell migration, spheroid formation and tumor growth (both *in vitro* and *in vivo*) in *STAT3* KO ovarian cancer cells. However, HEY cells displayed modest changes upon *STAT3* deletion. The HEY cell line was established from a human ovarian cancer xenograft (HX-62) and it is reported to be resistant to cis-platinum 43. Based on a molecular profile analysis of cell lines from The Cancer Cell Line Encyclopedia (CCLE), both HEY and SKOV3 cells have wild-type *TP53*, while OVCAR3 and OVCAR8 harbor *TP53* mutations, which are more likely to represent typical high-grade serous ovarian cancer (HGSOC) 44. In our study, high correlation (ρ > 0.93) was observed between the transcriptomes of SKOV3, OVCAR3 and OVCAR8 cells, indicating a high degree of global RNA expression similarity.

Wildtype ovarian cancer cells were used as control in this study. Transfection step in the gene editing process could result in subpopulation, which is inherently different than parental population. To check if the gene expression changes reported in this study could be caused by the enzymatic effect of Cas9 nuclease, we generated a Cas9 control cell line by transfecting SKOV3 cells with Cas9 nuclease mRNA and profiled these cells using RNA-seq and Bru-seq. Global steady state RNA profiles between WT and Cas9 control cell lines show a high degree of similarity by Spearman correlation (ρ=0.934) (Supplemental Figure S10). Eleven genes were found to change significantly in opposite directions. However, none of these eleven genes are genes that we are highlighting in our analysis related to *STAT3*.

To our knowledge, this study provides the first evidence that STAT3 regulates the expression of other STAT family members. To date, seven STAT family members have been identified in mammals. Gene knockout studies in mice have defined the specific function and biological importance of STAT members under normal conditions 45. However, the regulation and relationships between STAT family members are not well understood. Understanding the patterns of activation of these STATs in malignant cells and their effects on gene expression, have important implications for targeted cancer therapy. Several studies have shown that STAT3 and STAT5 both have oncogenic activities in many cancer types, but they are activated by different mechanisms and have distinct roles in tumor development. For example, activated STAT3 is expressed in invasive and metastatic tumors, while STAT5 is generally expressed in well-differentiated tumors 46. Notably, higher levels of activated STAT3 were detected in STAT5-deficient cultivated primary erythroblasts, suggesting that STAT3 compensates for a loss of STAT5 47. We observed that the expression of STAT5 is dependent on STAT3, as *STAT3* KO resulted in loss of STAT5 expression (especially STAT5α). Thus, our study demonstrated that STAT3 can modulate the expression of STAT5, and it is possible that STAT3 compensates for STAT5 signaling in ovarian cancer. Upon IL-2 stimulation, the IL-2 receptor recruits and phosphorylates STAT1, STAT3 and STAT5 through different interactions 48. Splenocytes from STAT5α KO display a partial defect in IL-2-induced proliferation due to impaired IL-2- mediated IL-2Rα expression 49. Deficiency of IL-2-induced proliferation is also observed in STAT3-deficient T cells, but not in STAT5β KO 50, 51. These findings may indicate that STAT3 and STAT5α are more dependent on IL-2 induced activation, while STAT5β has other biological effects. Indeed, we observed a greater loss of protein expression of STAT5α in *STAT3* KO cells in comparison with STAT5β. Thus, it is possible that the gene interaction between *STAT3* and *STAT5A* is tighter than *STAT3* and *STAT5B*. STAT1 and STAT3 have opposing biological effects in cancer progression: STAT3 is an oncoprotein, while STAT1 serves as a tumor suppressor 28. We identified that the transcription and expression of STAT1 is increased in *STAT3* KO ovarian cancer cells. gp130-linked IL-6 receptor can activate STAT1 efficiently when STAT3 is absent 52. In normal cells, it has also been reported that STAT1 and STAT3 are competitively activated by IFN-γ receptor subunit 1, and STAT3 could replace STAT1 to drive the transcription of genes downstream of STAT1 in STAT1-null mouse embryonic fibroblasts 28. Collectively, our data suggested that the expression of *STAT1* is significantly influenced by *STAT3* levels*.*

It is well known that STAT3 modulates cell growth and survival. Our data demonstrated that KO of *STAT3* resulted in reduced tumor growth in both 2D and 3D cell culture models and in xenograft models of ovarian cancer. Several recent studies reported that treatment of esophageal cancer cells with siSTAT3 resulted in G1 or G2/M phase arrest 53, 54. We observed that cells were enriched in G2/M phase after deletion of *STAT3* in ovarian cancer. After *STAT3* KO, enrichment of reduced transcriptional expression in the G2/M checkpoint hallmark gene set further supports this observation. Several studies have shown that aberrant STAT3 signaling promotes tumor proliferation by elevating the expression of *CCND1* (cyclin D1) 55 and inducing expression of anti-apoptotic proteins such as survivin 56, Mcl-1 57, c-Myc and Bcl-xL 12. We confirmed these observations and have identified several genes involved in cell cycle progression that were transcriptionally suppressed due to the deletion of *STAT3*. In addition, we observed a down regulation of E2F target genes as well as reduced E2F1 protein expression in *STAT3* KO cells. E2F controls cell cycle progression through transcriptional regulation of genes involved in cell cycle progression, checkpoint control, DNA replication and DNA repair pathways 58, 59. To our knowledge the connection between E2F and STAT3 has not been previously reported. Our data provide the first evidence of suppression on E2F signaling in response to loss of *STAT3*.

EMT is a complex process leading to the conversion of epithelial cells into mesenchymal cells. EMT can be induced by multiple extracellular signals that lead to the activation of a plethora of EMT-specific transcription factors. The EMT pathway plays an important role in all stages of cancer progression including cell morphology, motility, invasion, stemness, and drug resistance 60. Our genome-wide multi-omics findings support the hypothesis that STAT3 plays important roles in cell migration and extracellular matrix organization. Furthermore, our study presented a comprehensive molecular signature of EMT suppression upon *STAT3* deletion and highlighted the down-regulation of transcription factors involved in EMT, such as *SNAI2*, *ZEB1/2, TWIST1/2*, *ETS1* and *FOXC1*. These observations strongly suggest that STAT3 contributes to EMT progression in ovarian cancer. Additionally, a direct link between the EMT and cancer stem-like properties has been observed in several different tumor types 61. Induction of EMT in immortalized human mammary epithelial cells, by exposure to TGF-β or expression of Snail or Twist, is known to increase the ability of the tumor cells to form tumorspheres and acquire CD44^high^/CD24^low^ stemness markers 62. STAT3 is essential in maintaining undifferentiated mouse embryonic stem cells and has been shown to enhance cancer stemness properties 63. ALDH1 is a well-established stem cell marker. A recent study reported that over-expression of ALDH1A2 decreased cell growth and migration by down regulating STAT3 activation in ovarian cancer cells 64. However, the direct connection between STAT3 and other ALDH1A family members, ALDH1A1 and ALDH1A3, has not been established. In our study, we observed that deletion of *STAT3* inhibits spheroid formation capability and suppressed transcription of CD44 and ALDH1. Consistent with our observation, a previous study showed STAT3 is constitutively activated in ALDH^+^/CD44^+^/CD24^-^ stem cell-like triple-negative breast tumors, and inhibition of STAT3 by small molecule inhibitors could efficiently suppress tumorsphere-formation capabilities 65. Interestingly, the CD44^high^/CD24^low^ cells express high levels of mesenchymal markers and a low level of *CDH1* 62. Altogether, these observations imply that STAT3 promotes both EMT and cancer stemness as a transcription factor and is likely a major driving force in promoting metastasis in ovarian cancer.

STAT3 phosphorylation measurements were downloaded and analyzed from the TCGA ovarian cancer dataset in which STAT3 pY705 was quantified using the Reverse Phase Protein Array (RPPA) platform. No significant association was observed between phospho-STAT3 and ovarian cancer patient survival or disease progression (using stage and grade). Also, using a per-patient z-score transformation, we did not observe a significantly increased amount of phospho-STAT3 in the ovarian cancer cohort compared to other TCGA diseases. From a recent study of tissue microarrays in 341 ovarian patient samples, 95 cases (28%) were observed with positive expression of p-STAT3. Patients with negative p-STAT3 had significant improved overall survival compared with those with positive p-STAT3, though difference of progression-free survival was not statistically significant 16. In another study of patient ascites-derived ovarian cancer cells, 18 out of 20 patient samples were detected with high expression of p-STAT3 Tyr705, but not p-STAT3 Ser727 17.

In conclusion, our integrated analysis across multiple molecular profiling platforms revealed a complex molecular signaling network controlled by STAT3. Importantly, we demonstrated that STAT3 plays a critical role in cell-cycle progression, EMT, invasion, and maintenance of stemness in ovarian cancer in several model systems. For the first time, we identify a link between STAT3 and E2F target genes as well as alteration in STAT family members upon STAT3 deletion. An important therapeutic implication of our study is the dramatic differences between the effect of STAT3 deletion in 2D versus 3D and *in vivo*. STAT3 deletion causes remarkable tumor growth inhibition in several tumor xenograft models of ovarian cancer. These findings highlight the critical function of STAT3 in ovarian cancer tumor progression and provide new insights on its potential as a therapeutic target.

## Materials and Methods

### Antibodies and cell culture

Antibodies for E2F1(#3742), STAT3 (9132/ 9139), survivin (2808), and GAPDH (2118) were purchased from Cell Signaling Technology (Beverly, MA). Antibodies for STAT1 (464), STAT2 (514193), STAT4 (398228), STAT5α (271542), STAT5β (1656), STAT6 (374021), CDC2 p34 (54), CDK2 (6248), Chk1 (8408), CDC25C (13138), Plk (17783), MCM2 (373702), CDC45 (55569), cyclin E2 (28351), CDKN2AIP (81841), ALDH1A1 (374149), and actin (58673) were purchased from Santa Cruz Biotechnology (Dallas, TX). Antibodies for CD44 (MA5-13890) and ALDH1A3 (PA5-29188) were purchased from Thermo Fisher (Waltham, MA). Two individual siRNAs for STAT3 (29493 and 44275) were purchased from Santa Cruz Biotechnology (Dallas, TX).

OVCAR3, OVCAR8 and SKOV3 cells were purchased from National Cancer Institute, Bethesda, MD. HEY cells were kindly provided as a gift by Dr. Louis Dubeau (University of Southern California, CA). Mesenchymal stem cells were kindly provided as a gift by Dr. Karen McLean (University of Michigan, MI). All cells were grown as monolayers at 37°C in a humidified atmosphere of 5% CO_2_. All experiments were carried out using cells in the exponential growth phase. Cells were routinely checked for mycoplasma contamination with PlasmoTest (InvivoGen).

### CRISPR-Cas9 mediated genome editing

Three *STAT3* CRISPR guide sequences (1: ACAATCCGGGCAATCTCCAT, 2: CATTCGACTCTTGCAGGAAG, 3: GAAACTGCCGCAGCTCCATT) were designed and purchased from Invitrogen™, ThermoFisher Scientific. *In vitro* transcription of the gRNA template was carried out with the MEGAshortscript™ T7 Transcription Kit using the manufacturer's recommended conditions. The gRNA product was purified with the MEGAclearTM Transcription Clean-Up kit as described in the manual. RNA concentration was determined using a NanoDrop spectrophotometer. The percentage of locus-specific indel formation was measured and selected using the GeneArt® Genomic Cleavage Selection Kit (Invitrogen™, ThermoFisher Scientific). Target sequences were cloned into a cleavage selection vector, which contains the orange fluorescence protein (OFP) as a reporter for fluorescence-based cell sorting. The percentage of OFP-positive cells indicates the cleavage activity of CRISPR-Cas9. CRISPR-Cas9 mediated genome editing was performed as previously reported 42. Cas9 control cells were generated by transfecting SKOV3 cells with Cas9 nuclease mRNA only. At 48 h post-transfection, the cells were harvested for analysis of genome modification efficiency using the GeneArt Genomic Cleavage Detection kit (Invitrogen™, ThermoFisher Scientific). Additionally, cells were analyzed by flow cytometry to identify the OFP-positive population and were sorted by FACS. A single clone isolation was further performed on sorted OFP-positive cells. The clones were screened by Western blot to assess STAT3 protein expression levels. *STAT3* sequence in KO cells was confirmed with a homozygous 1 bp deletion by Sanger sequencing.

### 3D spheroid assay

After trypsinization, cell dilutions were re-suspended in media containing 10% FBS and collagen (Stemcell Technologies) on ice. Cells were then plated in U-bottomed 96-well plates (Corning) at 3000 cell/well with 0.015 mg/well collagen. After 24 h, single or multiple viable spheroids were generated. On Day 2, 4 and 6, spheroids were imaged by fluorescence microscopy (Olympus Corporation) using a 10× objective. Cell viability was measured with the CellTiter-Glo® 3D Cell Viability Assay (Promega), following the manufacturer's recommendations. Luminescence was measured at room temperature using a Synergy H1 Hybrid Multi-Mode Microplate Reader (BioTek Instruments, Inc).

### Cell proliferation assay

Cells were plated at 1 × 10^4^ cells/ well in 6-well plates. Triplicate wells were harvested by trypsinization and counted on Day 2, 4, 6 and 8. Data was analyzed using GraphPad Prism, and doubling times were calculated from the growth curves.

### Wound healing assay

Cells were seeded in 12-well plates (2 × 10^5^ cells/well) in RPMI or DMEM supplemented with 1% FBS overnight. The following day, a single scratch was made in each well. After 24 h, cells were fixed with 100% methanol for 10 min, stained with Giemsa nuclear stain (10% Giemsa, 10% methanol, and 80% distilled water) for 30 min at room temperature and washed with water. Stained cells were imaged via microscopy (Olympus Corporation) using a 10× objective. Data presented is a representative image of at least three independent experiments.

### Bru-Seq analysis of nascent RNA synthesis

Bru-Seq analysis was performed as previously reported 21, 22. Briefly, when cells reached 80-90% confluence, bromouridine (final concentration of 2 mM) was added to the media to label newly synthesized nascent RNA for 30 min. Cells were collected in TRIzol (Invitrogen) and total RNA was isolated. Bru-labeled, nascent RNA was isolated, converted into cDNA libraries and sequenced using an Illumina HiSeq 2000 sequencer (San Diego, CA). Sequencing reads were mapped to the hg38 reference sequence. Ensemble gene identifiers were mapped to HGNC symbols and Entrez identifiers using Gencode v27 annotations 66. Only measurements mapping to Entrez identifiers were considered and gene changes with absolute fold change > 2 and mean RPKM > 0.5 were considered significant.

### Protein identification and relative quantitation by TMT labeling and LC-Tandem MS

Cells were lysed with cell lysis buffer at 4 °C for 30 min and centrifuged (12,000 rpm, 10 min, 4 °C). Protein concentrations of supernatants were measured with the BCA assay (Thermo Fisher Scientific). Tandem Mass Tag (TMT) labeling was performed using the TMT-6plex™ isobaric labeling kit (ThermoFisher Scientific) according to the manufacturer's protocol with minor modifications. Briefly, 75 μg of protein from each sample was reduced with DTT (5 mM) at 45 °C for 1 h followed by alkylation with 2-chloroacetamide (15 mM) at room temperature for 30 min. Proteins were precipitated by adding 6 volumes of ice-cold acetone and incubated overnight at -20 °C. Precipitated proteins were pelleted by centrifugation (8000 × g, 10 min, 4 °C) and supernatant was discarded. The pellet was re-suspended in 100 μl of 100 mM TEAB and the digested overnight at 37 °C by adding 1.5 μg of trypsin (Promega). TMT reagents were reconstituted in 41 μl of anhydrous acetonitrile and digested peptides were transferred to the TMT reagent vial and incubated at room temperature for 1 h. The reaction was quenched by adding 8 μl of 5% hydroxylamine and incubated for 15 min. The samples were combined and dried. Prior to MS analysis, two dimensional separation of the samples was performed. For the first dimension, an offline fractionation of an aliquot of each sample mix (200 μg) using high pH reverse phase fractionation kit into 10 fractions was performed following the manufacturer's protocol (Pierce, Cat #84868). Fractions were dried and reconstituted in 12 μl of loading buffer (0.1% formic acid and 2% acetonitrile). In order to ensure quantitation accuracy, the MultiNotch-MS3 method was employed 67. MS was performed on the Orbitrap Fusion Tribrid with ETD (ThermoFisher) equipped with nano-LC system (Dionex RSLC-nano). UniProt accessions were mapped to HGNC gene symbols and Entrez identifiers using biomaRt v2.36.1 68. An absolute fold-change cut-off of 2 was used to label protein changes as significantly different between *STAT3* KO and parental SKOV3 protein read-outs. An absolute fold change > 2 was applied for ORA with all Entrez genes as a background universe.

### RNA-Seq Profiling

RNA sequencing of SKOV3 was profiled twice using two different controls: *STAT3* KO vs parental and *STAT3* KO vs Cas9 control. Main SKOV3 results described here are based on Cas9 control. RNA sequencing of OVCAR3 and OVCAR8 are based on parental controls.

Cells were lysed with TRIzol® Reagent (ThermoFisher Scientific) at room temperature. RNA was further purified with DirectZol kit (Zymo Research, Irvine, CA). RNA quality was assessed using the TapeStation (Agilent Technologies, Santa Clara, CA). Samples with RINs (RNA Integrity Numbers) of 8 or greater were prepared with TruSeq Stranded mRNA Library Prep (Illumina) per the supplier's protocol with 1μg of RNA and 12 cycles of PCR amplification. Libraries were checked for size on the TapeStation and quantified using the Kapa Biosystems library quantification kit (Illumina). The libraries were barcoded, pooled and sequenced on the HiSeq 4000 at the University of Michigan DNA Sequencing Core using 50bp single-end 50bp (OVCAR3 and OVCAR8) and paired-end 50bp (SKOV3) sequencing. Reads were mapped to GRCh38 using STAR v2.5.2 69 and gene quantifications were calculated using Cufflinks v2.2.1 70 to quantify refGene annotations. Gene read counts calculated using featureCounts 71 v1.6.1 were used to evaluate differential expression using DESeq2 v1.18.1 72. For OVCAR3 and OVCAR8, genes were considered significantly differentially expressed with a mean FPKM > 0.5 and absolute fold change > 1.5 and FDR adjusted *p*-value < 0.05. For SKOV3, genes were considered significantly differentially expressed with mean FPKM > 0.5 and absolute log2 fold change > 1.5 and FDR adjusted *p*-value < 0.05. All gene readouts where required to be mappable to both an HGNC and Entrez identifier to be considered for gene set enrichment analyses. This data has been deposited in NCBI's Gene Expression Omnibus 73 and are accessible through GEO Series accession number GSE134375 (https://www.ncbi.nlm.nih.gov/geo/query/acc.cgi?acc=GSE134375).

### Western blot

Cells were lysed with cell lysis buffer at 4 °C for 30 min and centrifuged (12 000 rpm, 10 min, 4 °C). Protein concentrations of supernatants were measured with BCA assay (ThermoFisher Scientific). 30 μg of protein per sample was subjected to SDS-PAGE analysis and electro-transferred to methanol-activated immobilon-FL PVDF membranes (EMD Millipore, Billerica, MA). Membranes were blocked with 5% milk in TBST buffer and probed with primary antibodies with dilutions according to the manufacturer's instructions, subsequently with Dylight 800-conjugated secondary antibodies (ThermoFisher Scientific). The fluorescent signal was detected using the Odyssey Imaging System (LI-COR Biosciences).

### *In vivo* tumor xenograft studies

4-6-week old athymic mice, NOG (NOD/Shi-*scid*/IL-2Rγ^null^) mice and humanized immune huNOG mice (all from Taconic Biosciences, Inc.) were used for *in vivo* studies. Mice were randomly grouped (*N* = 5) to generate cell line-derived xenografts. HEY and HEY *STAT3* KO (3 × 10^6^ cells), OVCAR3, OVCAR3 *STAT3* KO, OVCAR8, OVCAR8 *STAT3* KO, SKOV3 and SKOV3 *STAT3* KO cells (5 × 10^6^ cells) were implanted subcutaneously into the dorsal flanks of athymic or NSG mice under aseptic conditions as described 74. For MSCs co-culture experiment, 3 × 10^6^ SKOV3 or SKOV3 *STAT3* KO cells were co-cultured with or without 2.5 × 10^5^ MSCs. Cells were harvested next day and were implanted subcutaneously into the dorsal flanks of randomly grouped NSG mice (*N* = 3). Tumor size was monitored twice a week by caliper measurement using the following equation: tumor volume (mm^3^) = D × *d*^2^/2, where D and *d* are the longest and shortest diameters, respectively. Study was concluded when tumor size reached at least 800 mm^3^.

### Statistical and bioinformatics analysis

For Bru-Seq, RNA-Seq, and proteomic functional enrichment, gene set enrichment of differentially expressed genes was performed using DAVIDWebService v1.14 75 with a background of all measured protein coding genes. Gene sets with an FDR adjusted *p*-value < 0.1 were considered significant. GSEA v2.2.3 was used with v6 gene sets sourced from MSigDB using 10 000 permutations in weighted mode. Genes with mean expression > 0.5 FPKM were ranked by fold change and gene sets with FDR adjusted *p*-values < 0.05 were considered significantly enriched.

All experiments were repeated with at least 3-5 biological replicates except Bru-Seq analysis. Un-paired Student's t-test was used for statistical analysis, and two-tailed *p* value was determined with GraphPad Prism 7.0. *** indicate a *p*-value < 0.001, ** indicate a *p*-value < 0.01, and * indicate a *p*-value < 0.05. All data is represented as mean ± standard error of the mean.

## Supplementary Material

Supplementary figures and tables.

## Abbreviations

Bru-Seq: bromouridine sequencing
DAVID: the database for annotation, visualization and integrated discovery
EMT: epithelial to mesenchymal transition
FDR: false discovery rate
GBMLGG: glioblastoma and lower grade glioma
GSEA: gene set enrichment analysis
KIRC: kidney renal clear cell carcinoma
KO: knockout
MS: mass spectrometry
ORA: over-representation analysis
OV: ovarian
RNA-Seq: RNA sequencing
RPKM: reads per kilobase of transcript per million mapped reads
TCGA: the cancer genome atlas
UVM: uveal melanoma
WT: wildtype
