# Supplementary material for: Multi-omics profiling reveals key signaling pathways in ovarian cancer controlled by STAT3
Source: Theranostics. 2019 Jul 28;9(19):5478–96. doi: 10.7150/thno.33444 (PMC6735387; doi:10.7150/thno.33444)
Supplement: Supplementary file 1 — Supplementary figures and tables. [file thnov09p5478s1.pdf]

Supplemental section

Multi-omics profiling reveals key signaling pathways in ovarian cancer controlled by STAT3

Tiangong Lu, Armand Bankhead III, Mats Ljungman, and Nouri Neamati

Figure legends

Figure S1: Generation of *STAT3* KO ovarian cancer cell lines using CRISPR-Cas9 genome editing. Cells were transfected with 3 different *STAT3* gRNAs, Cas9 nuclease mRNA and cleavage selection vectors. Flow cytometry analysis of OFP-positive cells showing percentages for each guide RNA in (A). SKOV3, (B). OVCAR3, OVCAR8 and HEY cells using guide RNAs for the *STAT3* gene 72 hours post-transfection. STAT3 protein expression for each gRNA was detected by Western blot. (C). No STAT3 protein expression was detected in single cell cloned *STAT3* KO ovarian cancer cells. NFC = normalized fold change.

Figure S2: Characterization of *STAT3* KO ovarian cancer cell lines. (A). Migration capability of WT and *STAT3* KO cells was determined by a wound-healing assay. Bars from each cell line indicate wound area at 0h and 24h in percentage normalized to control. (B). Cell viability of spheroids at Days 2, 4 and 6 of WT and *STAT3* KO cells in 3D spheroid assay. Luminescence representing cell viability was measured using the CellTiter-Glo® 3D Cell Viability Assay. Error bars indicate mean ± SEM. and $**p < 0.01$, $***p < 0.001$.

Figure S3: STAT3 KO inhibits tumor growth in mouse xenograft models. Image of tumors in nude mice (n = 5) injected with SKOV3, HEY, OVCAR3 and OVCAR8 WT/ STAT3 KO cells.

Figure S4: Overlap of Gene Set Enrichment Analysis (GSEA) enriched gene sets for *STAT3* knockout in three ovarian cancer cell lines (A) Common gene sets are listed with gene sets enriched in SKOV3 multi-omic analysis highlighted in bold.

Figure S5: KM plots of genes having significant associations with patient survival analyzed from TCGA.

Figure S6: clueGO Visualization of Functional Enrichment of 41 genes

Figure S7: *STAT3* KO results in downregulation of genes involved in epithelial-mesenchymal transition. (A). Heatmap shows log2 fold change of *STAT3* knockout versus parental cell lines across Bru-Seq, RNA-Seq, and proteomic platforms. Black boxes indicate genes with significant differential expression (as described in methods). (B) Boxplots represents log2 fold change average of genes regulating epithelial and mesenchymal phenotypes in SKOV3, OVCAR3 and OVCAR8 *STAT3* KO/WT identified by RNA-seq. ** indicate a *p*-value < 0.05, NS, not significant, unpaired Student's t-test, two-tailed *p*-value.

Figure S8: *STAT3* KO results in downregulation of genes involved in cell cycle and alters expression of STAT family members. (A). The G2/M Checkpoint hallmark gene set was significantly down-regulated as determined using GSEA in SKOV3 and OVCAR3 cell lines. FDR adjusted *p*-value in SKOV3 Bru-Seq, RNA-Seq, proteomics and OVCAR3 RNA-Seq, are <0.001, 0.05, 0.01, and 0.01, respectively. Colors represent log2 fold change between *STAT3* KO cell lines and parental cell lines with black boxes highlighting genes that were significantly different as described in Methods. (B). Key cell cycle mediators were suppressed in *STAT3* KO cells. Protein expression levels were determined by Western blot. (C). Fold change of RNA and protein expression levels of STAT family members in WT/ *STAT3* KO cells. ND, not detected.

Figure S9: 50 significant STAT3 co-expression gene sets in common between 4 diseases.

(A) Significant associations with high STAT3 mRNA expression and reduced survival were identified in GMBLGG, KIRP and UVM. (B) STAT3 co-expression modelled using Gene Set Enrichment Analysis (GSEA) to identify gene sets enriched for genes correlated with STAT3 expression in ovarian (OV), glioblastoma and lower grade glioma (GBMLGG), uveal melanoma (UVM), and kidney renal clear cell carcinoma (KIRC) TCGA cohorts. 50 gene sets were in

common across all four disease cohorts and are listed to the left with orange stars highlighting JAK/STAT related gene sets and yellow stars highlighting gene sets related to cytokine activity.

Figure S10: Comparison of SKOV3 log2 fold changes using parental SKOV3 control versus parental SKOV treated with CASP9 control. All points shown correspond to genes with at least 0.5 meanFPKM expression and FDR adjusted p-value < 0.05. Dashed lines represent log2 FC +/- 1. Nine genes were identified switching directions due to using different controls (|log2 FC| > 2): C3, FDFT1, FGFBP1, ID3, INSIG1, KISS1, MVK, TRIM22, OASL.

Supplemental Figure S1

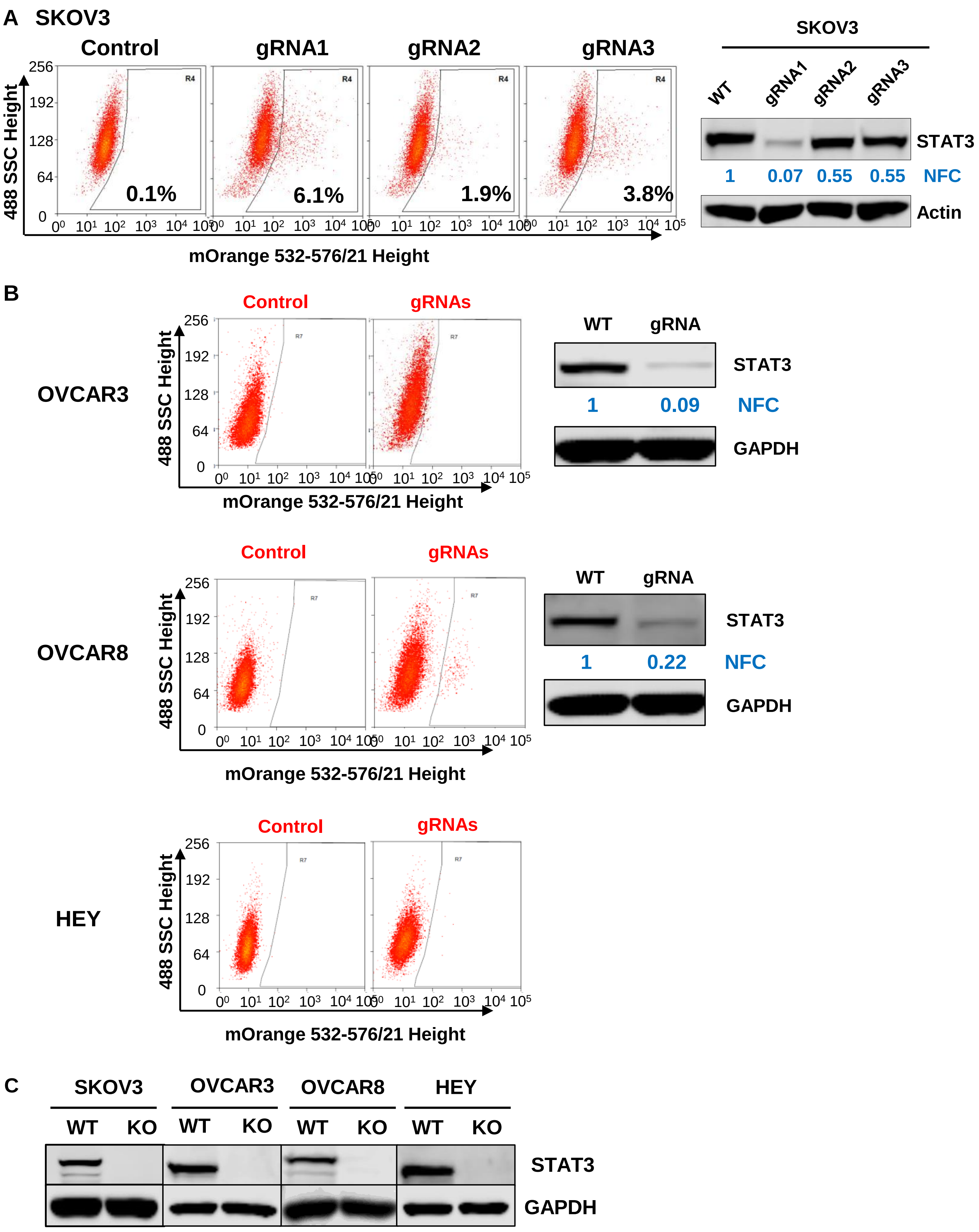

Supplemental Figure S2

A

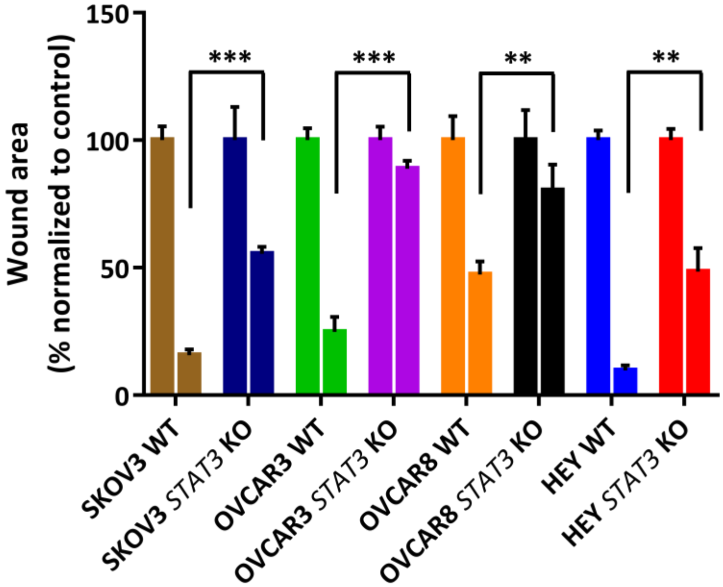

B

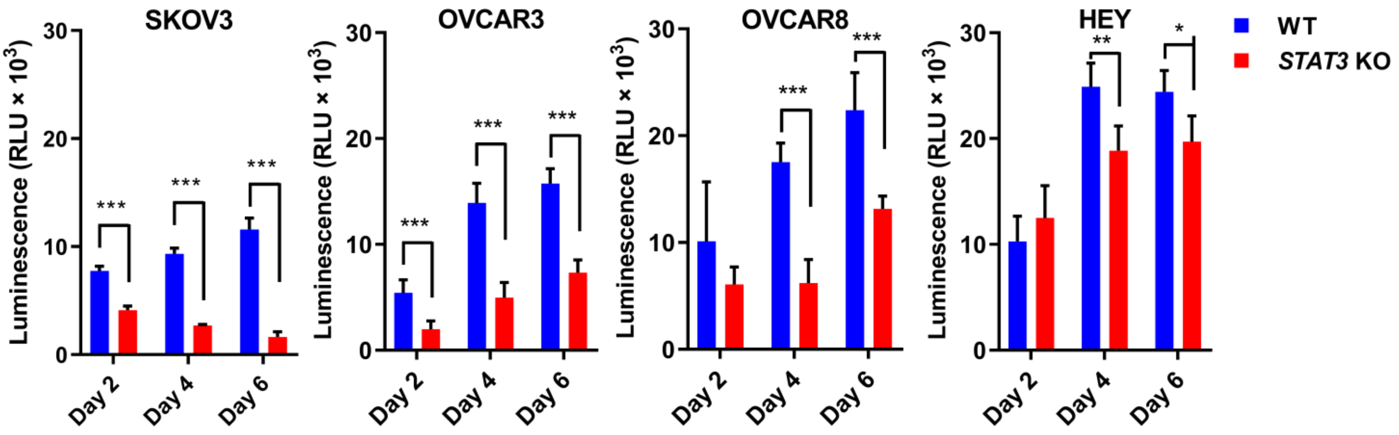

Supplemental Figure S3

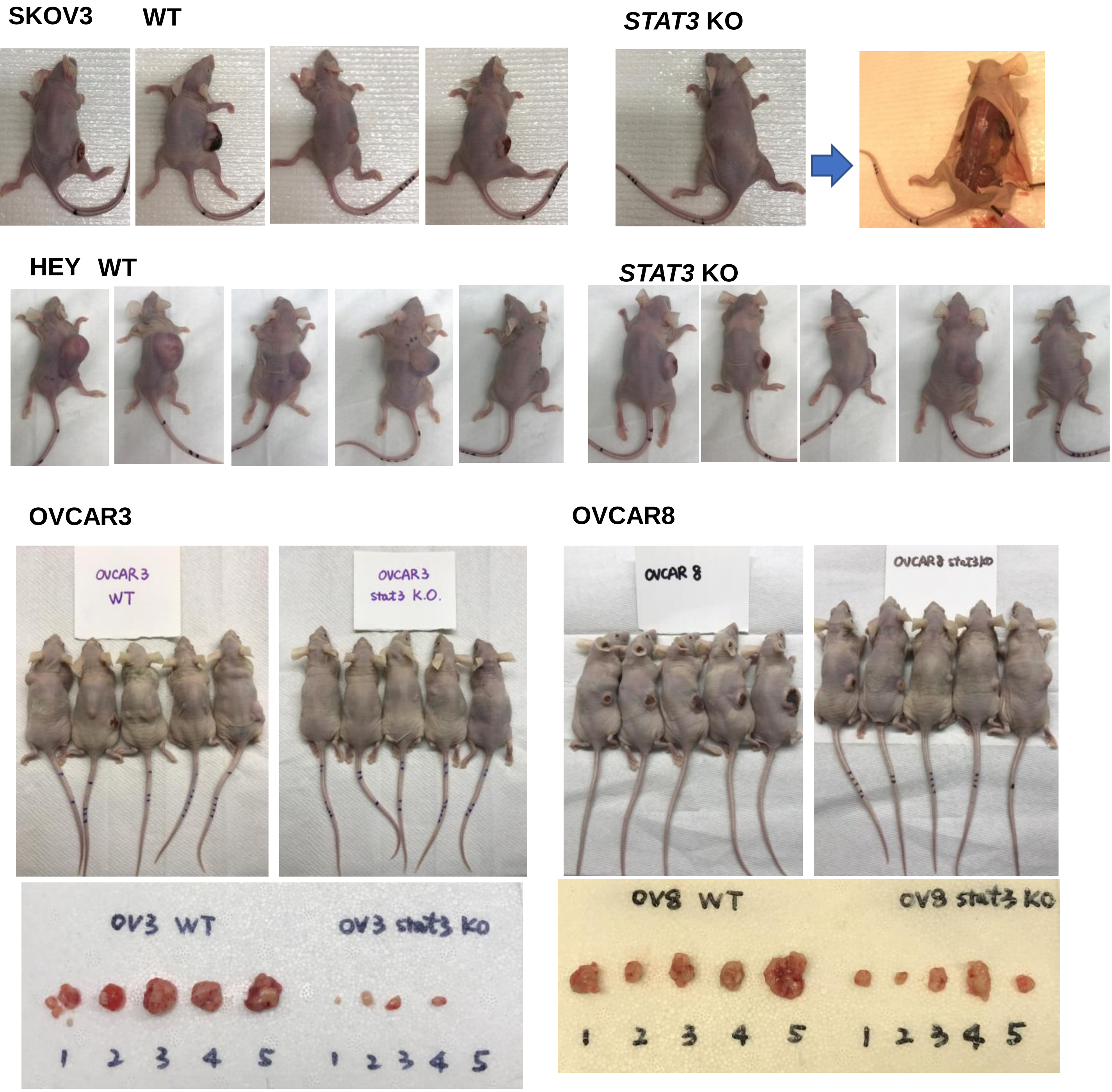

Supplemental Figure S4

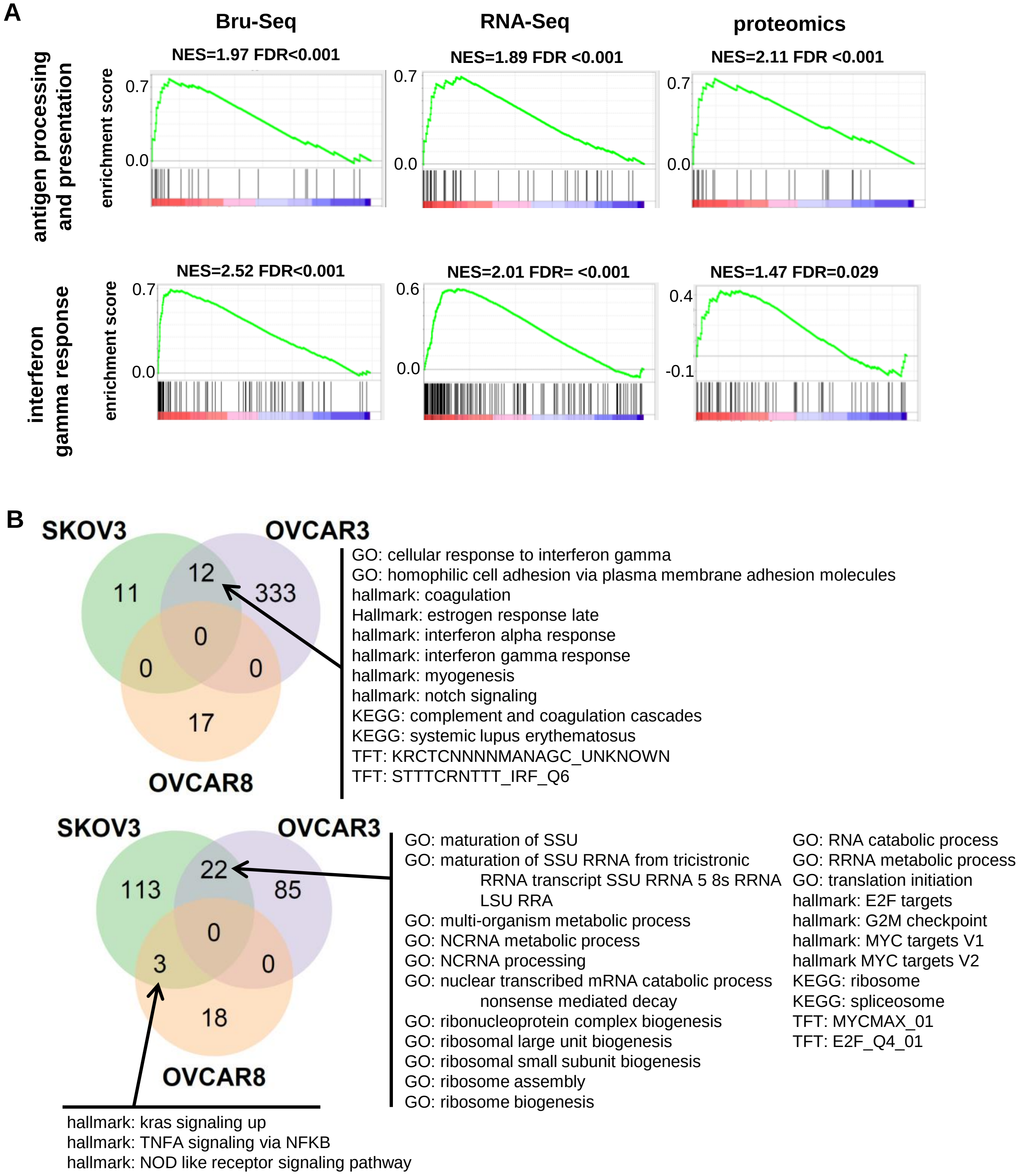

Supplemental Figure S5

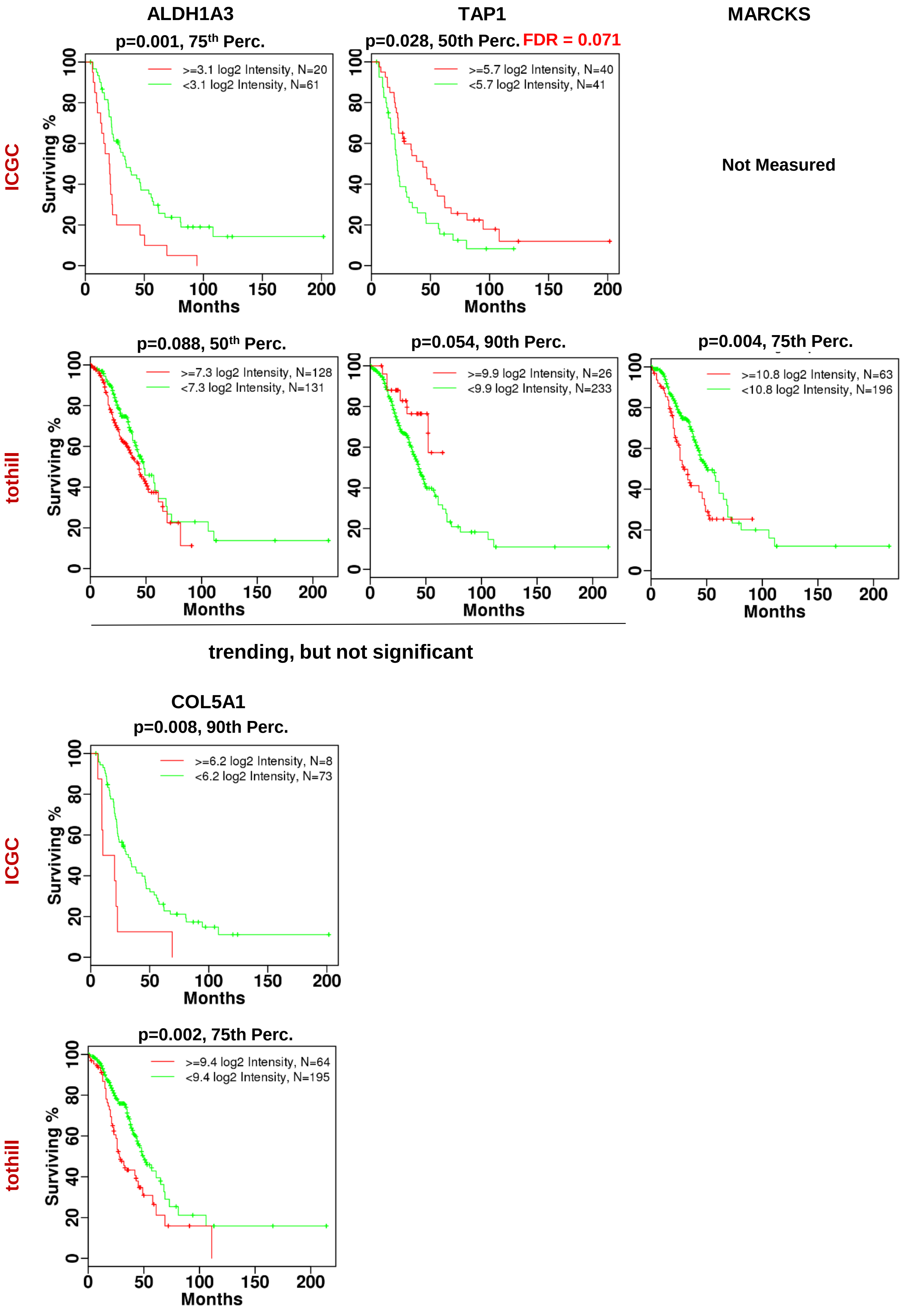

Supplemental Figure S6

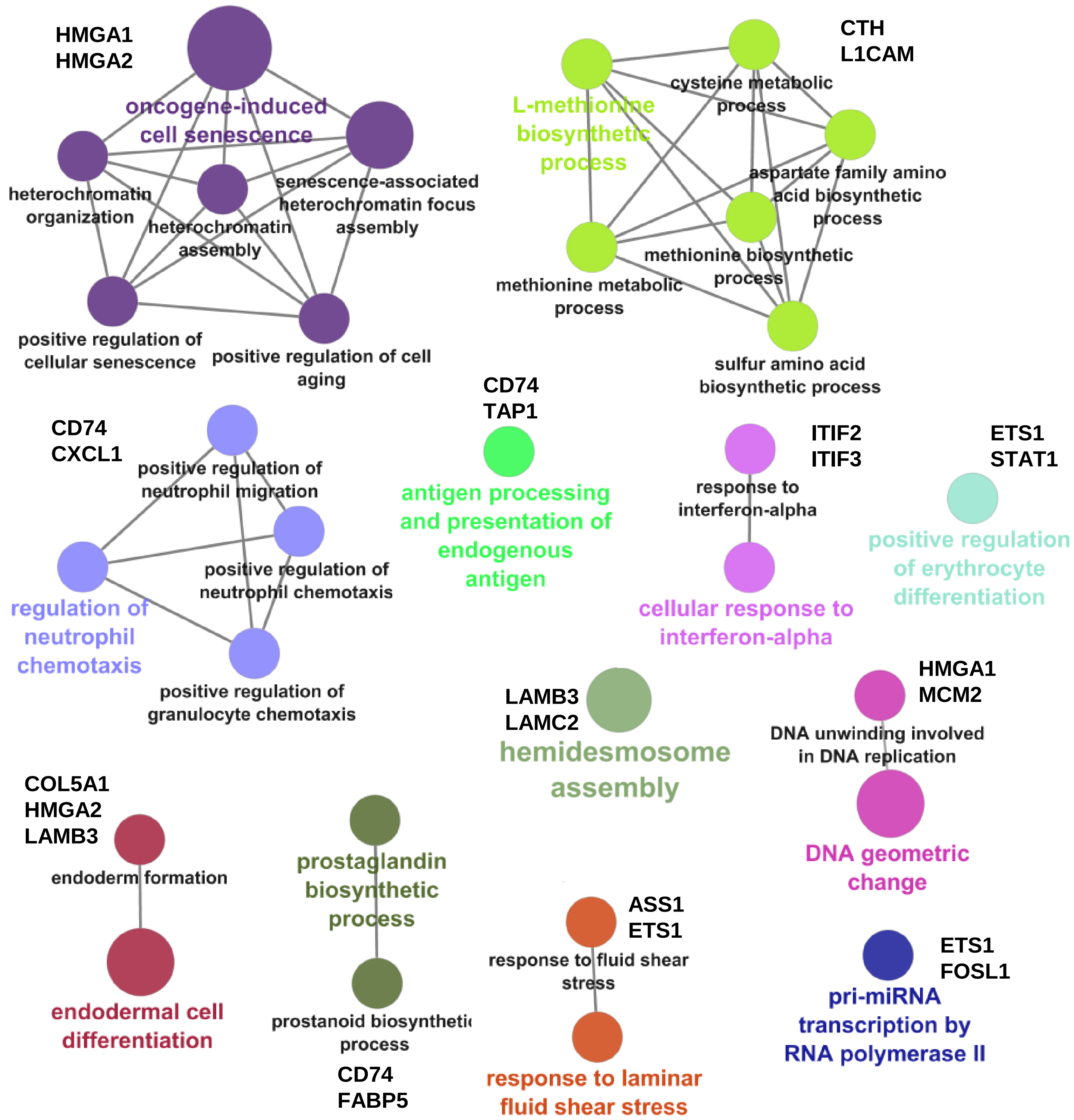

Supplemental Figure S7

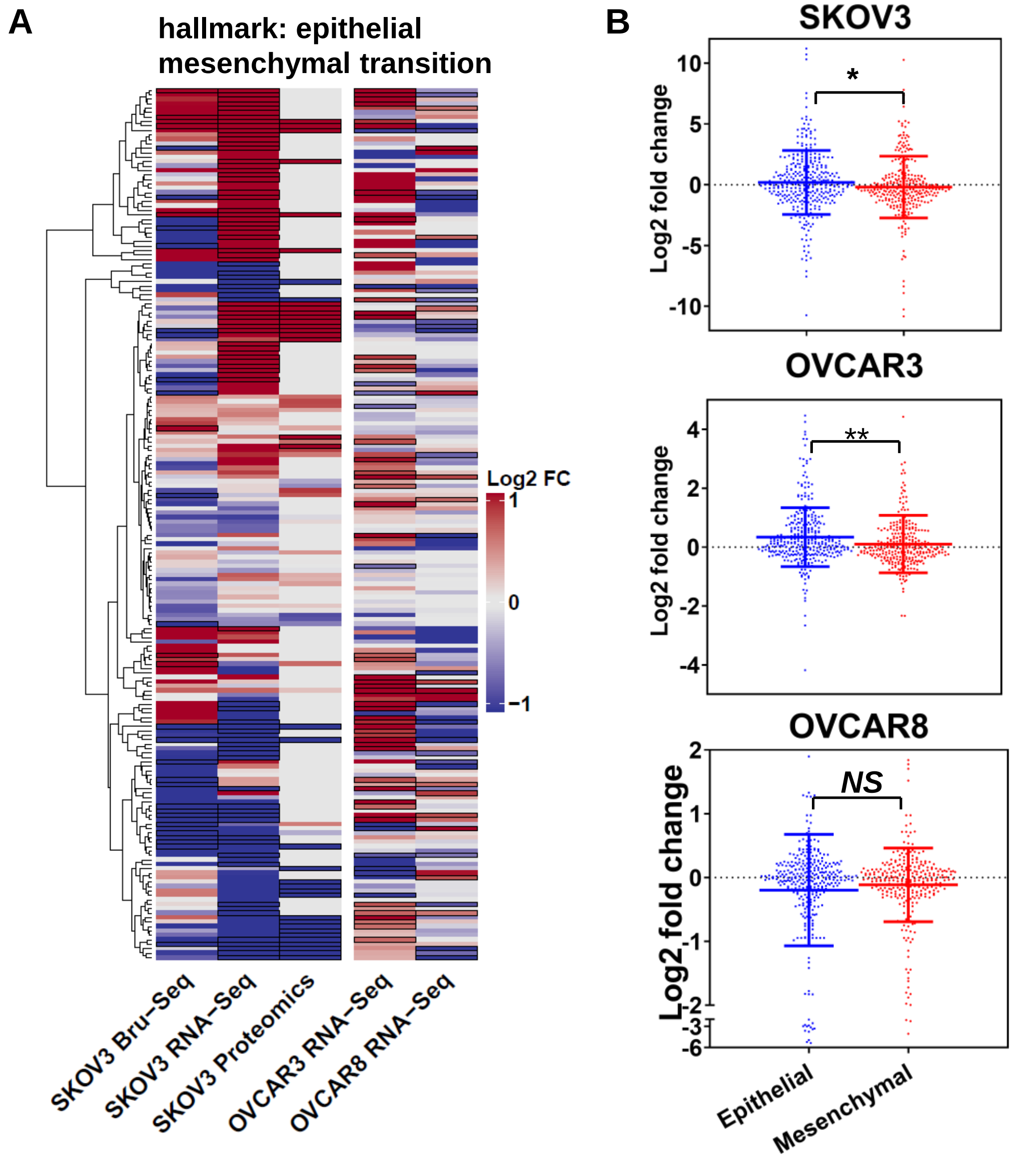

Supplemental Figure S8

A hallmark: G2M checkpoint

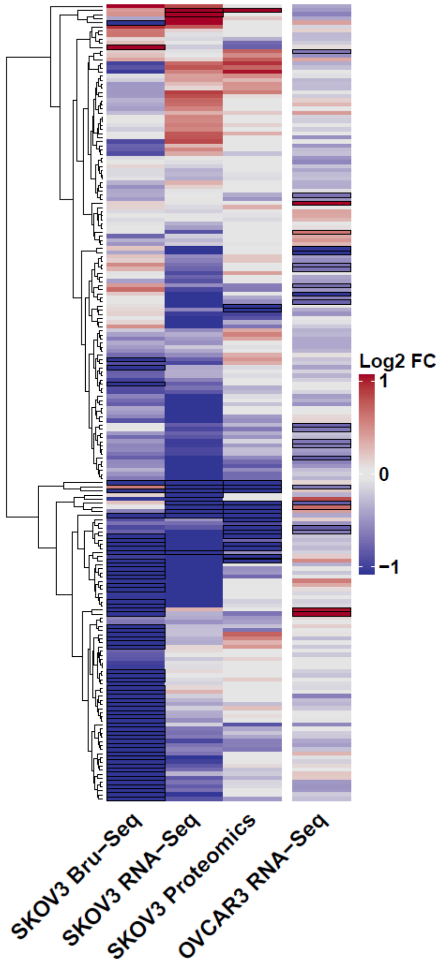

B

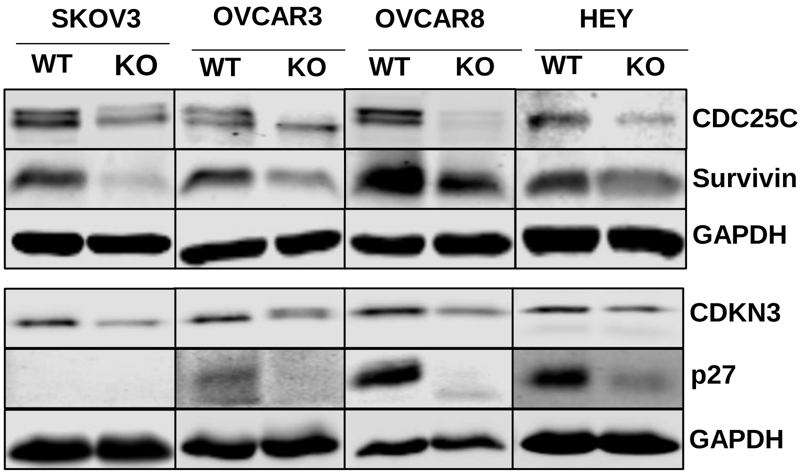

C

	SKOV3 Bru-seq	SKOV3 Proteomics	SKOV3 RNA-seq	OVCAR3 RNA-seq	OVCAR8 RNA-seq
STAT1	5.78	2.17	10.63	-1.11	1.09
STAT2	4.55	ND	7.47	1.44	1.11
STAT3	-1.05	-5.75	-2.53	-2.98	-1.62
STAT4	-1.51	ND	-1.0	10.56	-1.28
STAT5A	-5.35	ND	-17.70	1.08	-1.56
STAT5B	-1.14	1.02	1.05	-1.33	-1.16
STAT6	1.68	-1.03	ND	ND	ND

Supplemental Figure S9

A

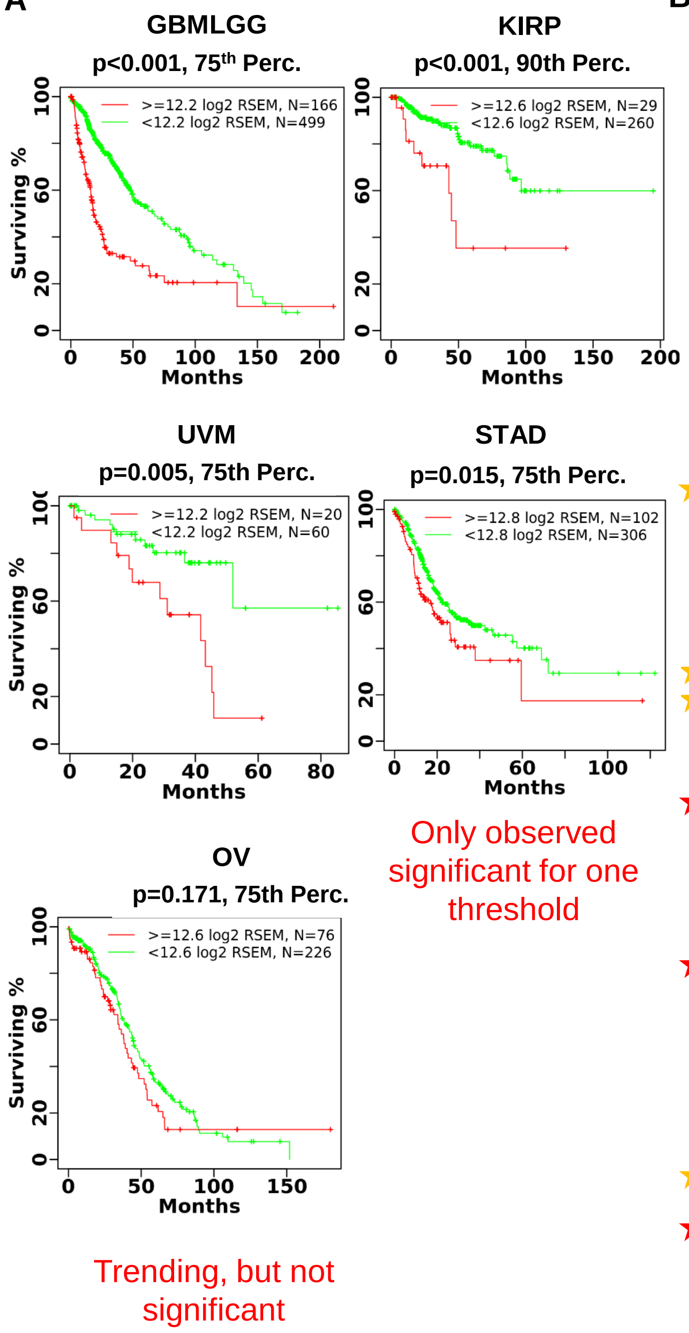

B **50 significant gene sets in common between 4 diseases**

GO: activation of immune response
GO: adaptive immune response
GO: adaptive immune response based on somatic recombination of immune receptors built from immunoglobulin superfamily domains
GO: cellular response to interferon gamma
GO: immune effector process
GO: inflammatory response
GO: interferon gamma mediated signaling pathway
GO: leukocyte activation
GO: leukocyte cell cell adhesion
GO: leukocyte differentiation
GO: leukocyte migration
GO: lymphocyte activation
GO: lymphocyte differentiation
GO: positive regulation of cell activation
GO: positive regulation of cell adhesion
GO: positive regulation of cell cell adhesion
★ GO: positive regulation of cytokine production
GO: positive regulation of immune response
GO: regulation of adaptive immune response
GO: regulation of b cell activation
GO: regulation of b cell proliferation
GO: regulation of cell activation
GO; regulation of cell cell adhesion
★ GO: regulation of cytokine biosynthetic process
★ GO: regulation of cytokine secretion
GO: regulation of homotypic cell cell adhesion
GO: regulation of leukocyte differentiation
GO: response to molecule of bacterial origin
★ GO: stat cascade
GO: T cell differentiation
hallmark: allograft rejection
hallmark: apoptosis
hallmark: complement
hallmark: IL2 STAT5 signaling
★ hallmark: IL6 JAK STAT3 signaling
hallmark: inflammatory response
hallmark: interferon alpha response
hallmark: interferon gamma response
hallmark: KRAS signaling up
hallmark: TNFA signaling via NFKB
KEGG: apoptosis
KEGG: cell adhesion molecules CAMs
★ KEGG: cytokine cytokine receptor interaction
KEGG: hematopoietic cell lineage
★ KEGG: JAK STAT signaling pathway
KEGG: leishmania infection
KEGG: natural kill cell mediated cytotoxicity
KEGG: NOD like receptor signaling pathway
KEGG: toll like receptor signaling pathway
KEGG: viral myocarditis

Supplemental Figure S10

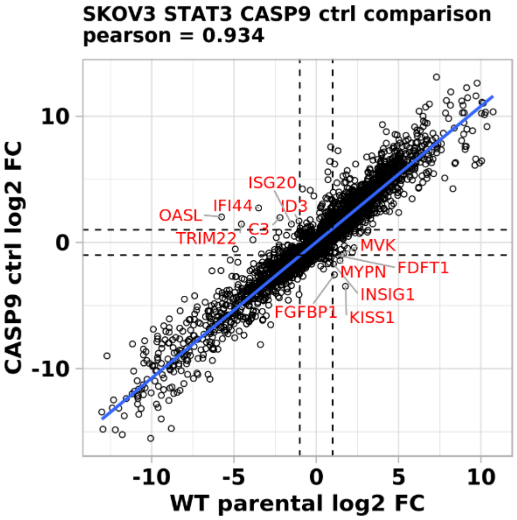

Table S1. Summary of STAT3-deficiency phenotypes

Species and models	Genome modification	Phenotypes and findings
Ovarian cancer cell A2780	STAT3 overexpression (OE)	Develop larger tumor and distant metastases invading the mesentery, peritoneum and the liver in mice[1]
Mouse myeloid leukemic M1 cells	dominant-negative STAT3 mutant (STAT3DN)	No induction of differentiation response to IL-6 or LIF, indicating STAT3 activation is essential for IL-6 or LIF-mediated growth arrest and differentiation of M1 cells.[2]
mouse pro-B cell line BAF-B03	STAT3DN	STAT3DN did not proliferate and underwent cell death accompanied by DNA fragmentation, indicating STAT3 transmits an anti-apoptotic signal.[3]
Ovarian cancer cell CAOV3	short hairpin RNA (shRNA)	Inhibits the cell growth *in vitro*. Expressions of Bcl-xL, cyclin D1, and c-myc were down-regulated, whereas the cleaved caspase 3 was up-regulated.[4]
Ovarian cancer cell A2780R	shRNA	Cell growth and wound healing decline. Expression of several genes responsible for cell survival, migration and invasion (STAT3, VEGF, cyclin D2, Akt, c-myc, ATF2 and surviving) [1]
Ovarian cancer cell SKOV3	siRNA	Suppress cell growth, arrest the cell cycle in G1 phase and induce massive apoptosis. Expressions of BcL-2, cyclin D1, and c-Myc were suppressed.[5]
Ovarian cancer cell OVCAR3	siRNA	Expression of cyclin D1, survivin, and VEGF in ovarian cancer cells were down-regulated both at transcription and translation levels. Suppress cell growth and induce massive apoptosis *in vitro*.[6]
Human Cutaneous T-cell lymphomas cell Hut78	siRNA	Induced apoptotic cell death. Moreover, STAT3 inhibition down-regulated the expression of Bcl2 family of anti-apoptotic gene Bcl-xL.[7]
Human astrocytes and astrocytoma cells	siRNA	Induces apoptosis in several astrocytoma cell lines, but not in primary human astrocytes. Down regulated expression of the survivin and Bcl-xL in the A172 glioblastoma cell line.[8]
Oral squamous cell carcinoma cells	siRNA	Decreased expression of CCND1 and VEGF[9]
Prostate cancer cells DU145	siRNA	Unphosphorylated form of STAT3 binds to regulatory regions of proapoptotic genes and prevents their expression in tumor cells but not normal cells.[10]
colorectal cancer cells SW480s	siRNA	26 and 21 known miRNAs were significantly overexpressed and down-expressed. “Pathways in cancer” is top one[11]
Mouse Breast Tumor (4T1 cells) in Immunocompetent Mice	shRNA	c-Myc expression was reduced 75% in cells, activated Src was virtually eliminated but not the level of the Src protein. Expression of Twist protein was eliminated in STAT3 knockdown cells. Invasion activity was strongly inhibited. The proliferation rate of cells in STAT3 knockdown cells was similar; the cell cycle was also not affected.[12]
SCC-3 cells	shRNA	Suppressed cell proliferation *in vitro* and *in vivo*. Level of c-MYC, cyclin D1, and survivin were down-regulated<br>ATP5B and latexin were up-regulated, and ALDOA, 15-HPGD and serpin B4 was down-regulated<br>G6P, F6P, F6BP, and the ATP/ADP ratio were shown to decrease in STAT3 shRNA-treated cells. In contrast, alanine and lactate increased in shRNA-treated cells. PDK1, PFKFB3, PGM1 and PKM were down-regulated[13]
Mice	mice deficient in STAT3 by gene targeting	Targeted disruption of the mouse STAT3 gene leads to early embryonic lethality, which may be due to a defect in visceral endoderm function, such as nutritional insufficiency. [14]
Mice	Cre-LoxP methodology to generate mice with T cells-specific STAT3 knockout (KO)	STAT3-deficient T cells displayed a severely impaired proliferative response, demonstrating the anti-apoptotic function of STAT3[15]

Mice	Cre-LoxP methodology to generate muscle-specific STAT3 KO	STAT3 activation does not underlie the development of HFD-induced skeletal muscle insulin resistance.[16]
Mice	Cre-loxP recombination system to ablate STAT3 in cardiac myocytes	Leads to increased susceptibility to doxorubicin-induced heart failure and sensitivity to inflammation, cardiac fibrosis, and heart failure with advanced age[17]
Mice	Cre-LoxP methodology to generate liver-specific STAT3 KO	STAT3 is required for survival in the acute stage after 70% hepatectomy and plays a role in inflammatory reaction after hepatocyte necrosis. The hepatocytic STAT3 may have limited role in liver mass recovery although DNA synthesis may be impaired.[18]
Mice	Cre-LoxP methodology to generate Keratinocyte-specific STAT3 KO	STAT3 is essential to the second and subsequent hair cycles (skin remodeling) although it is dispensable for the first hair cycle (morphogenesis).[19]
Mice	Cre-LoxP methodology to generate macrophages and neutrophils-specific STAT3 KO	The mutant mice are highly susceptible to endotoxin shock with increased production of inflammatory cytokines such as TNF alpha, IL-1, IFN gamma, and IL-6.[20]
Mice embryonic stem cell	Cre recombination-mediated excision	an essential role for a critical amount of STAT3 in the maintenance of an undifferentiated ES cell phenotype<br>STAT3 function in ES cells is linked to the maintenance of a stem-cell phenotype independent of cell proliferation.<br>a minimal dose of STAT3 is required for ES cell propagation and pluripotency[21]
Mice	crossing $STAT3^{loxP}$ mice with Nes-Cre transgenic mice	Nes-STAT3−/− : limited migration and resulted in markedly widespread infiltration of inflammatory cells, neural disruption and demyelination with severe motor deficits after contusive spinal cord injury (SCI).<br>STAT3 is a key regulator of reactive astrocytes in the healing process after SCI[22]

Reference

1 Saini, U. *et al.* Elevated STAT3 expression in ovarian cancer ascites promotes invasion and metastasis: a potential therapeutic target. *Oncogene* **36**, 168-181, doi:10.1038/onc.2016.197 (2017).

2 Minami, M. *et al.* STAT3 activation is a critical step in gp130-mediated terminal differentiation and growth arrest of a myeloid cell line. *Proc Natl Acad Sci U S A* **93**, 3963-3966 (1996).

3 Fukada, T. *et al.* Two signals are necessary for cell proliferation induced by a cytokine receptor gp130: involvement of STAT3 in anti-apoptosis. *Immunity* **5**, 449-460 (1996).

4 Huang, F., Tong, X., Fu, L. & Zhang, R. Knockdown of STAT3 by shRNA inhibits the growth of CAOV3 ovarian cancer cell line in vitro and in vivo. *Acta Biochim Biophys Sin (Shanghai)* **40**, 519-525 (2008).

5 Zhao, S. H. *et al.* Knockdown of stat3 expression by RNAi inhibits in vitro growth of human ovarian cancer. *Radiol Oncol* **45**, 196-203, doi:10.2478/v10019-011-0013-8 (2011).

6 Cai, L. *et al.* Growth inhibition of human ovarian cancer cells by blocking STAT3 activation with small interfering RNA. *Eur J Obstet Gynecol Reprod Biol* **148**, 73-80, doi:10.1016/j.ejogrb.2009.09.018 (2010).

7 Verma, N. K., Davies, A. M., Long, A., Kelleher, D. & Volkov, Y. STAT3 knockdown by siRNA induces apoptosis in human cutaneous T-cell lymphoma line Hut78 via

downregulation of Bcl-xL. *Cell Mol Biol Lett* **15**, 342-355, doi:10.2478/s11658-010-0008-2 (2010).
8 Konnikova, L., Kotecki, M., Kruger, M. M. & Cochran, B. H. Knockdown of STAT3 expression by RNAi induces apoptosis in astrocytoma cells. *BMC Cancer* **3**, 23, doi:10.1186/1471-2407-3-23 (2003).
9 Klosek, S. K., Nakashiro, K., Hara, S., Goda, H. & Hamakawa, H. Stat3 as a molecular target in RNA interference-based treatment of oral squamous cell carcinoma. *Oncol Rep* **20**, 873-878 (2008).
10 Timofeeva, O. A. *et al.* STAT3 suppresses transcription of proapoptotic genes in cancer cells with the involvement of its N-terminal domain. *Proc Natl Acad Sci U S A* **110**, 1267-1272, doi:10.1073/pnas.1211805110 (2013).
11 Zhang, J., Luo, X., Li, H., Deng, L. & Wang, Y. Genome-wide uncovering of STAT3-mediated miRNA expression profiles in colorectal cancer cell lines. *Biomed Res Int* **2014**, 187105, doi:10.1155/2014/187105 (2014).
12 Ling, X. & Arlinghaus, R. B. Knockdown of STAT3 expression by RNA interference inhibits the induction of breast tumors in immunocompetent mice. *Cancer Res* **65**, 2532-2536, doi:10.1158/0008-5472.CAN-04-2425 (2005).
13 Akiyama, Y. *et al.* Effect of STAT3 inhibition on the metabolic switch in a highly STAT3-activated lymphoma cell line. *Cancer Genomics Proteomics* **12**, 133-142 (2015).
14 Takeda, K. *et al.* Targeted disruption of the mouse Stat3 gene leads to early embryonic lethality. *Proc Natl Acad Sci U S A* **94**, 3801-3804 (1997).
15 Takeda, K. *et al.* Stat3 activation is responsible for IL-6-dependent T cell proliferation through preventing apoptosis: generation and characterization of T cell-specific Stat3-deficient mice. *J Immunol* **161**, 4652-4660 (1998).
16 White, A. T., LaBarge, S. A., McCurdy, C. E. & Schenk, S. Knockout of STAT3 in skeletal muscle does not prevent high-fat diet-induced insulin resistance. *Mol Metab* **4**, 569-575, doi:10.1016/j.molmet.2015.05.001 (2015).
17 Jacoby, J. J. *et al.* Cardiomyocyte-restricted knockout of STAT3 results in higher sensitivity to inflammation, cardiac fibrosis, and heart failure with advanced age. *Proc Natl Acad Sci U S A* **100**, 12929-12934, doi:10.1073/pnas.2134694100 (2003).
18 Moh, A. *et al.* Role of STAT3 in liver regeneration: survival, DNA synthesis, inflammatory reaction and liver mass recovery. *Lab Invest* **87**, 1018-1028, doi:10.1038/labinvest.3700630 (2007).
19 Sano, S. *et al.* Keratinocyte-specific ablation of Stat3 exhibits impaired skin remodeling, but does not affect skin morphogenesis. *EMBO J* **18**, 4657-4668, doi:10.1093/emboj/18.17.4657 (1999).
20 Takeda, K. *et al.* Enhanced Th1 activity and development of chronic enterocolitis in mice devoid of Stat3 in macrophages and neutrophils. *Immunity* **10**, 39-49 (1999).
21 Raz, R., Lee, C. K., Cannizzaro, L. A., d'Eustachio, P. & Levy, D. E. Essential role of STAT3 for embryonic stem cell pluripotency. *Proc Natl Acad Sci U S A* **96**, 2846-2851 (1999).
22 Okada, S. *et al.* Conditional ablation of Stat3 or Socs3 discloses a dual role for reactive astrocytes after spinal cord injury. *Nat Med* **12**, 829-834, doi:10.1038/nm1425 (2006).

Table S2. Top 25 upregulated and downregulated genes_*STAT3* KO/WT

cellLine	Gene	dataType	Entrez	Fold Change	log2 Fold Change
SKOV3	COL15A1	Bru-seq	1306	714.5711	9.480934
SKOV3	ALDH1A1	Bru-seq	216	484.5644	8.920545
SKOV3	RSAD2	Bru-seq	91543	279.1468	8.12488
SKOV3	GDAP1L1	Bru-seq	78997	240.1323	7.907686
SKOV3	PRDM16	Bru-seq	63976	234.5478	7.873738
SKOV3	NTRK2	Bru-seq	4915	229.0439	7.83948
SKOV3	DEFB1	Bru-seq	1672	206.6254	7.690874
SKOV3	POU5F1B	Bru-seq	5462	195.4564	7.610703
SKOV3	CNN1	Bru-seq	1264	195.4564	7.610703
SKOV3	LCN2	Bru-seq	3934	189.8719	7.568883
SKOV3	TNNC1	Bru-seq	7134	178.7029	7.481419
SKOV3	ALX4	Bru-seq	60529	171.5291	7.422309
SKOV3	KCNJ5	Bru-seq	3762	167.5339	7.38831
SKOV3	CD74	Bru-seq	972	161.9494	7.3394
SKOV3	ZNF462	Bru-seq	58499	152.3074	7.250842
SKOV3	CD33	Bru-seq	945	139.6115	7.125274
SKOV3	HRK	Bru-seq	8739	134.0414	7.066535
SKOV3	BEST1	Bru-seq	7439	134.027	7.066379
SKOV3	TUBB2B	Bru-seq	347733	134.027	7.066379
SKOV3	COL9A2	Bru-seq	1298	128.4425	7.004978
SKOV3	PLA1A	Bru-seq	51365	117.2734	6.873733
SKOV3	DUSP15	Bru-seq	128853	117.2734	6.873733
SKOV3	PSD	Bru-seq	5662	111.6889	6.803343
SKOV3	DIRAS1	Bru-seq	148252	111.6889	6.803343
SKOV3	CEACAM20	Bru-seq	125931	100.6691	6.653478
SKOV3	SCN2A	Bru-seq	6326	-2813.16	-11.458
SKOV3	KYNU	Bru-seq	8942	-1401.52	-10.4528
SKOV3	HTATIP2	Bru-seq	10553	-1333.15	-10.3806
SKOV3	LRRN1	Bru-seq	57633	-710.503	-9.4727
SKOV3	ZNF311	Bru-seq	282890	-645.434	-9.33413
SKOV3	DCAF12L1	Bru-seq	139170	-454.985	-8.82968
SKOV3	SLC14A1	Bru-seq	6563	-415.606	-8.69907
SKOV3	ZNF785	Bru-seq	146540	-407.372	-8.6702
SKOV3	SHROOM4	Bru-seq	57477	-391.772	-8.61387
SKOV3	PCDH18	Bru-seq	54510	-365.05	-8.51195
SKOV3	ACCS	Bru-seq	84680	-354.469	-8.46952
SKOV3	PKIA	Bru-seq	5569	-350.737	-8.45424
SKOV3	CDH18	Bru-seq	1016	-325.129	-8.34487
SKOV3	ESM1	Bru-seq	11082	-301.566	-8.23633
SKOV3	HIST1H3I	Bru-seq	8354	-259.242	-8.01816

SKOV3	FGF5	Bru-seq	2250	-231.655	-7.85583
SKOV3	G0S2	Bru-seq	50486	-211.675	-7.72571
SKOV3	UGT8	Bru-seq	7368	-208.624	-7.70476
SKOV3	PCDH9	Bru-seq	5101	-192.488	-7.58862
SKOV3	FOXP2	Bru-seq	93986	-189.098	-7.56299
SKOV3	XKR8	Bru-seq	55113	-179.924	-7.49124
SKOV3	ZNF22	Bru-seq	7570	-155.748	-7.28307
SKOV3	NRGN	Bru-seq	4900	-137.589	-7.10422
SKOV3	HAVCR1	Bru-seq	26762	-119.077	-6.89575
SKOV3	RAB34	Bru-seq	83871	-111.129	-6.79609
SKOV3	COL1A2	RNA-seq	1278	8802.138	13.10364
SKOV3	PDZK1	RNA-seq	5174	6320.761	12.62588
SKOV3	JPH3	RNA-seq	57338	4908.364	12.26103
SKOV3	IGFL3	RNA-seq	388555	3899.117	11.92893
SKOV3	ATP10A	RNA-seq	57194	3134.783	11.61415
SKOV3	SLC12A3	RNA-seq	6559	2831.174	11.46718
SKOV3	C11orf86	RNA-seq	254439	2427.722	11.24539
SKOV3	ROS1	RNA-seq	6098	2366.761	11.2087
SKOV3	ALX4	RNA-seq	60529	2209.125	11.10926
SKOV3	CACNG4	RNA-seq	27092	2101.662	11.03731
SKOV3	FABP3	RNA-seq	2170	2001.957	10.9672
SKOV3	SHC2	RNA-seq	25759	1888.888	10.88332
SKOV3	TRPC4	RNA-seq	7223	1878.033	10.87501
SKOV3	ACKR3	RNA-seq	57007	1866.115	10.86582
SKOV3	FIBCD1	RNA-seq	84929	1748.225	10.77168
SKOV3	MMP13	RNA-seq	4322	1692.223	10.7247
SKOV3	BEGAIN	RNA-seq	57596	1353.7	10.40269
SKOV3	CD4	RNA-seq	920	1304.972	10.3498
SKOV3	TNNT2	RNA-seq	7139	1277.572	10.31919
SKOV3	TMEM178B	RNA-seq	1.01E+08	1238.41	10.27427
SKOV3	COL1A1	RNA-seq	1277	1237.968	10.27376
SKOV3	ANXA8	RNA-seq	653145	1222.251	10.25532
SKOV3	ALDH1A1	RNA-seq	216	1200.049	10.22888
SKOV3	TOX	RNA-seq	9760	1113.605	10.12102
SKOV3	LRRN2	RNA-seq	10446	1028.974	10.00699
SKOV3	ARHGDIB	RNA-seq	397	-47101.2	-15.5235
SKOV3	G0S2	RNA-seq	50486	-38373.1	-15.2278
SKOV3	ESM1	RNA-seq	11082	-29439.8	-14.8455
SKOV3	AEBP1	RNA-seq	165	-28521.4	-14.7998
SKOV3	ECSCR	RNA-seq	641700	-27291.6	-14.7362
SKOV3	ADAMTS12	RNA-seq	81792	-16190.2	-13.9828
SKOV3	GPX3	RNA-seq	2878	-13683	-13.7401
SKOV3	CDH5	RNA-seq	1003	-12581.7	-13.619

SKOV3	CHRDL1	RNA-seq	91851	-11079.1	-13.4356
SKOV3	LAYN	RNA-seq	143903	-9179.38	-13.1642
SKOV3	ENG	RNA-seq	2022	-8961.54	-13.1295
SKOV3	ARMCX2	RNA-seq	9823	-8874.43	-13.1154
SKOV3	MT1E	RNA-seq	4493	-8683.71	-13.0841
SKOV3	ANKFY1	RNA-seq	51479	-7212.06	-12.8162
SKOV3	RAB34	RNA-seq	83871	-7151.8	-12.8041
SKOV3	SLC6A15	RNA-seq	55117	-5888.89	-12.5238
SKOV3	SHANK1	RNA-seq	50944	-5707.08	-12.4785
SKOV3	CDH4	RNA-seq	1002	-5287.55	-12.3684
SKOV3	IL13RA2	RNA-seq	3598	-5270.13	-12.3636
SKOV3	EDA2R	RNA-seq	60401	-4740.47	-12.2108
SKOV3	NCAM1	RNA-seq	4684	-4445.62	-12.1182
SKOV3	AJAP1	RNA-seq	55966	-4401.95	-12.1039
SKOV3	CACNA2D4	RNA-seq	93589	-4280.23	-12.0635
SKOV3	ZNF22	RNA-seq	7570	-4084.86	-11.9961
SKOV3	UCHL1	RNA-seq	7345	-4011.31	-11.9699
OVCAR3	ZNF429	RNA-seq	353088	352.1973	8.46024
OVCAR3	MAGEB2	RNA-seq	4113	133.4066	7.059686
OVCAR3	SERPINB2	RNA-seq	5055	125.1308	6.967294
OVCAR3	HTR2C	RNA-seq	3358	89.51497	6.484057
OVCAR3	CCNA1	RNA-seq	8900	86.66481	6.437374
OVCAR3	ALPPL2	RNA-seq	251	79.75243	6.317457
OVCAR3	PLA2G4D	RNA-seq	283748	63.26414	5.983316
OVCAR3	FOXJ1	RNA-seq	2302	59.39025	5.892154
OVCAR3	ERVFRD-1	RNA-seq	405754	57.59086	5.847768
OVCAR3	RAB17	RNA-seq	64284	54.84366	5.777253
OVCAR3	CSF2RB	RNA-seq	1439	54.11576	5.757977
OVCAR3	OLR1	RNA-seq	4973	49.48238	5.628843
OVCAR3	CXCL1	RNA-seq	2919	48.23039	5.591871
OVCAR3	CLEC11A	RNA-seq	6320	45.70341	5.51423
OVCAR3	SPP1	RNA-seq	6696	43.90986	5.456473
OVCAR3	NPHS1	RNA-seq	4868	42.64501	5.414305
OVCAR3	SIGLEC6	RNA-seq	946	41.89596	5.388739
OVCAR3	CGA	RNA-seq	1081	36.49465	5.189613
OVCAR3	ANKFN1	RNA-seq	162282	36.02418	5.170894
OVCAR3	NAT2	RNA-seq	10	36.00096	5.169963
OVCAR3	PSG2	RNA-seq	5670	35.48392	5.149093
OVCAR3	SUN3	RNA-seq	256979	34.86157	5.123566
OVCAR3	PLEKHG7	RNA-seq	440107	33.82226	5.079901
OVCAR3	RNASE7	RNA-seq	84659	33.34591	5.059438
OVCAR3	RHCE	RNA-seq	6006	32.69215	5.030872
OVCAR3	SPIN4	RNA-seq	139886	-2029.07	-10.9866

OVCAR3	CCDC152	RNA-seq	1E+08	-225.403	-7.81636
OVCAR3	NF2	RNA-seq	4771	-216.667	-7.75933
OVCAR3	ADAMTS2	RNA-seq	9509	-156.285	-7.28803
OVCAR3	SLC19A3	RNA-seq	80704	-90.4594	-6.4992
OVCAR3	PAK3	RNA-seq	5063	-88.7943	-6.47239
OVCAR3	WIPF3	RNA-seq	644150	-74.4303	-6.21782
OVCAR3	LRRC34	RNA-seq	151827	-56.7357	-5.82619
OVCAR3	VAT1L	RNA-seq	57687	-41.9161	-5.38943
OVCAR3	NCAM1	RNA-seq	4684	-41.5357	-5.37628
OVCAR3	LRAT	RNA-seq	9227	-38.9284	-5.28275
OVCAR3	RTL8B	RNA-seq	441518	-34.0307	-5.08877
OVCAR3	CFI	RNA-seq	3426	-33.4373	-5.06338
OVCAR3	IFNLR1	RNA-seq	163702	-33.2358	-5.05467
OVCAR3	EPHA8	RNA-seq	2046	-31.2055	-4.96373
OVCAR3	C2orf88	RNA-seq	84281	-31.1644	-4.96183
OVCAR3	ARHGDIG	RNA-seq	398	-30.7629	-4.94312
OVCAR3	PDIA2	RNA-seq	64714	-28.9608	-4.85603
OVCAR3	ZFP69	RNA-seq	339559	-28.6177	-4.83883
OVCAR3	GALNT5	RNA-seq	11227	-25.7187	-4.68474
OVCAR3	DCDC2	RNA-seq	51473	-24.2924	-4.60243
OVCAR3	BCHE	RNA-seq	590	-22.0451	-4.46239
OVCAR3	ZNF718	RNA-seq	255403	-19.1707	-4.26083
OVCAR3	ACADL	RNA-seq	33	-18.1534	-4.18217
OVCAR3	CELF2	RNA-seq	10659	-17.1648	-4.10138
OVCAR8	SOX11	RNA-seq	6664	34.47162	5.107337
OVCAR8	ALG1L2	RNA-seq	644974	25.45038	4.669615
OVCAR8	CFAP58	RNA-seq	159686	25.32691	4.662599
OVCAR8	SLC26A7	RNA-seq	115111	23.58551	4.559829
OVCAR8	HEATR4	RNA-seq	399671	20.79551	4.3782
OVCAR8	ACRV1	RNA-seq	56	20.49474	4.357182
OVCAR8	SYNDIG1L	RNA-seq	646658	18.46685	4.206866
OVCAR8	CFAP161	RNA-seq	161502	16.77318	4.068084
OVCAR8	TRIM69	RNA-seq	140691	16.47844	4.042508
OVCAR8	PXT1	RNA-seq	222659	16.38715	4.034493
OVCAR8	NR2E3	RNA-seq	10002	14.67364	3.875155
OVCAR8	ATP6V1C2	RNA-seq	245973	14.46255	3.85425
OVCAR8	TAGLN3	RNA-seq	29114	14.37366	3.845356
OVCAR8	NEURL1	RNA-seq	9148	12.39375	3.631541
OVCAR8	MPP4	RNA-seq	58538	11.21669	3.487575
OVCAR8	IL36G	RNA-seq	56300	10.7676	3.428625
OVCAR8	CFAP221	RNA-seq	200373	10.28759	3.362834
OVCAR8	CDHR3	RNA-seq	222256	10.25628	3.358436
OVCAR8	SHBG	RNA-seq	6462	9.502857	3.248361

OVCAR8	BTN1A1	RNA-seq	696	9.388465	3.230889
OVCAR8	CALHM3	RNA-seq	119395	9.355788	3.225859
OVCAR8	DACH2	RNA-seq	117154	9.304395	3.217912
OVCAR8	MEFV	RNA-seq	4210	9.277685	3.213765
OVCAR8	CCL25	RNA-seq	6370	9.003893	3.170549
OVCAR8	IQCF1	RNA-seq	132141	8.942137	3.16062
OVCAR8	TENM2	RNA-seq	57451	-354.717	-8.47053
OVCAR8	PTPN20	RNA-seq	26095	-250.652	-7.96954
OVCAR8	SSPO	RNA-seq	23145	-219.338	-7.77701
OVCAR8	GPR65	RNA-seq	8477	-171.608	-7.42297
OVCAR8	SCIN	RNA-seq	85477	-146.959	-7.19927
OVCAR8	LAMP5	RNA-seq	24141	-140.848	-7.13799
OVCAR8	ABO	RNA-seq	28	-133.105	-7.05642
OVCAR8	CCL20	RNA-seq	6364	-111.671	-6.80311
OVCAR8	ACP5	RNA-seq	54	-110.115	-6.78287
OVCAR8	MAP10	RNA-seq	54627	-99.8346	-6.64147
OVCAR8	DNAJA4	RNA-seq	55466	-97.506	-6.60742
OVCAR8	GPR4	RNA-seq	2828	-92.5024	-6.53142
OVCAR8	C6orf141	RNA-seq	135398	-79.6456	-6.31552
OVCAR8	IL13RA2	RNA-seq	3598	-70.8621	-6.14694
OVCAR8	ZNF492	RNA-seq	57615	-62.0255	-5.95479
OVCAR8	IL10RA	RNA-seq	3587	-59.3334	-5.89077
OVCAR8	DGKG	RNA-seq	1608	-56.5412	-5.82123
OVCAR8	SYPL2	RNA-seq	284612	-53.5918	-5.74394
OVCAR8	BDKRB1	RNA-seq	623	-49.5738	-5.63151
OVCAR8	ANXA8L1	RNA-seq	643650	-46.8105	-5.54876
OVCAR8	DEUP1	RNA-seq	159989	-44.923	-5.48938
OVCAR8	PRRX2	RNA-seq	51450	-43.8688	-5.45512
OVCAR8	TPSG1	RNA-seq	25823	-41.7049	-5.38215
OVCAR8	WT1	RNA-seq	7490	-41.2664	-5.3669
OVCAR8	HCLS1	RNA-seq	3059	-38.8989	-5.28166
SKOV3	SPANXD	Proteomics	64648	54.407	5.77
SKOV3	UGT1A6	Proteomics	54578	24.044	4.59
SKOV3	SNCG	Proteomics	6623	19.745	4.3
SKOV3	TAGLN	Proteomics	6876	17.274	4.11
SKOV3	DCDC2	Proteomics	51473	16.78	4.07
SKOV3	CDH1	Proteomics	999	16.417	4.04
SKOV3	SERPINB9	Proteomics	5272	16.073	4.01
SKOV3	ALPP	Proteomics	250	15.766	3.98
SKOV3	PDZK1	Proteomics	5174	14.569	3.86
SKOV3	CRYAB	Proteomics	1410	13.765	3.78
SKOV3	SPANXB1	Proteomics	728695	13.267	3.73
SKOV3	RCN3	Proteomics	57333	13.147	3.72

SKOV3	EPB41L1	Proteomics	2036	12.797	3.68
SKOV3	S100A4	Proteomics	6275	12.49	3.64
SKOV3	L1CAM	Proteomics	3897	12.505	3.64
SKOV3	SDC2	Proteomics	6383	12.231	3.61
SKOV3	HLA-B	Proteomics	3106	11.266	3.49
SKOV3	FABP3	Proteomics	2170	10.922	3.45
SKOV3	FOLR1	Proteomics	2348	10.502	3.39
SKOV3	DPYSL5	Proteomics	56896	9.718	3.28
SKOV3	DHRS2	Proteomics	10202	9.159	3.2
SKOV3	KRT5	Proteomics	3852	8.819	3.14
SKOV3	HSPB1	Proteomics	3315	8.663	3.11
SKOV3	COL5A1	Proteomics	1289	8.526	3.09
SKOV3	SULT1A4	Proteomics	445329	8.275	3.05
SKOV3	MAST4	Proteomics	375449	-100	-6.64
SKOV3	ALOX5AP	Proteomics	241	-100	-6.64
SKOV3	PDLIM4	Proteomics	8572	-100	-6.64
SKOV3	FBRSL1	Proteomics	57666	-100	-6.64
SKOV3	ARHGDIB	Proteomics	397	-52.6316	-5.75
SKOV3	CLEC11A	Proteomics	6320	-47.619	-5.6
SKOV3	SLC43A3	Proteomics	29015	-37.037	-5.19
SKOV3	DOK1	Proteomics	1796	-23.2558	-4.56
SKOV3	MARCKS	Proteomics	4082	-22.2222	-4.48
SKOV3	UCHL1	Proteomics	7345	-21.7391	-4.43
SKOV3	SMARCC1	Proteomics	6599	-21.2766	-4.42
SKOV3	UQCRH	Proteomics	7388	-18.5185	-4.22
SKOV3	KRT7	Proteomics	3855	-17.8571	-4.16
SKOV3	ACAA2	Proteomics	10449	-17.8571	-4.16
SKOV3	KCTD12	Proteomics	115207	-16.129	-4
SKOV3	TGM2	Proteomics	7052	-12.987	-3.71
SKOV3	CXCL1	Proteomics	2919	-12.8205	-3.69
SKOV3	KCNAB1	Proteomics	7881	-12.6582	-3.66
SKOV3	HCLS1	Proteomics	3059	-11.4943	-3.52
SKOV3	ECSCR	Proteomics	641700	-10.989	-3.45
SKOV3	FKBP10	Proteomics	60681	-10.6383	-3.4
SKOV3	FABP5	Proteomics	2171	-10.6383	-3.41
SKOV3	ENG	Proteomics	2022	-10.2041	-3.35
SKOV3	FLNC	Proteomics	2318	-9.43396	-3.24
SKOV3	HIST1H1B	Proteomics	3009	-9.25926	-3.2

Table S3. DAVID analysis top 15 genesets correlated with *STAT3* KO/WT

**cellLine**	**dataType**	**Source**	**geneSet**	**pValue**	**Number Genes**	**Total GeneSet Genes**
SKOV3	Bru-Seq	goBP	GO:0000819~sister chromatid segregation	1.98E-26	56	224
SKOV3	Bru-Seq	goBP	GO:0007059~chromosome segregation	1.72E-23	64	333
SKOV3	Bru-Seq	goBP	GO:0098813~nuclear chromosome segregation	9.7E-22	57	286
SKOV3	Bru-Seq	goBP	GO:0071103~DNA conformation change	5.49E-21	55	276
SKOV3	Bru-Seq	goBP	GO:0006323~DNA packaging	1.49E-19	45	198
SKOV3	Bru-Seq	goBP	GO:0007062~sister chromatid cohesion	5.96E-18	35	128
SKOV3	Bru-Seq	goBP	GO:0006260~DNA replication	3E-17	51	288
SKOV3	Bru-Seq	goBP	GO:0000070~mitotic sister chromatid segregation	1.56E-15	34	142
SKOV3	Bru-Seq	goBP	GO:0031497~chromatin assembly	1E-14	35	160
SKOV3	Bru-Seq	goBP	GO:0006261~DNA-dependent DNA replication	2E-14	33	145
SKOV3	Bru-Seq	goBP	GO:0006333~chromatin assembly or disassembly	7.29E-13	35	184
SKOV3	Bru-Seq	goBP	GO:0006302~double-strand break repair	3.16E-12	36	204
SKOV3	Bru-Seq	goBP	GO:0006334~nucleosome assembly	3.91E-12	30	144
SKOV3	Bru-Seq	goBP	GO:0034508~centromere complex assembly	6.01E-12	19	54
SKOV3	Bru-Seq	goBP	GO:0000075~cell cycle checkpoint	2.15E-11	37	229
SKOV3	Bru-Seq	kegg	hsa05206:MicroRNAs in cancer	1.58E-07	35	255
SKOV3	Bru-Seq	kegg	hsa04110:Cell cycle	2.01E-07	23	124
SKOV3	Bru-Seq	kegg	hsa03030:DNA replication	9.3E-07	12	36
SKOV3	Bru-Seq	kegg	hsa05034:Alcoholism	6.98E-06	25	175
SKOV3	Bru-Seq	kegg	hsa05322:Systemic lupus erythematosus	7.26E-06	21	130
SKOV3	Bru-Seq	kegg	hsa00240:Pyrimidine metabolism	4.16E-05	17	101
SKOV3	Bru-Seq	kegg	hsa00970:Aminoacyl-tRNA biosynthesis	0.000292	10	44
SKOV3	Bru-Seq	kegg	hsa04115:p53 signaling pathway	0.001844	11	67
SKOV3	Bru-Seq	kegg	hsa05203:Viral carcinogenesis	0.003381	21	204
SKOV3	Bru-Seq	kegg	hsa04612:Antigen processing and presentation	0.006313	10	67
SKOV3	Bru-Seq	kegg	hsa00670:One carbon pool by folate	0.016503	5	20
SKOV3	Bru-Seq	kegg	hsa05166:HTLV-I infection	0.018023	22	254

SKOV3	Bru-Seq	kegg	hsa00983:Drug metabolism - other enzymes	0.027473	7	46
SKOV3	Bru-Seq	kegg	hsa05202:Transcriptional misregulation in cancer	0.043562	15	167
SKOV3	Bru-Seq	kegg	hsa00053:Ascorbate and aldarate metabolism	0.045325	5	27
SKOV3	RNA-Seq	goBP	GO:0034340~response to type I interferon	2.82E-12	34	77
SKOV3	RNA-Seq	goBP	GO:0071357~cellular response to type I interferon	2.86E-12	33	73
SKOV3	RNA-Seq	goBP	GO:0060337~type I interferon signaling pathway	2.86E-12	33	73
SKOV3	RNA-Seq	goBP	GO:0030198~extracellular matrix organization	6.83E-11	81	333
SKOV3	RNA-Seq	goBP	GO:0043062~extracellular structure organization	7.97E-11	81	334
SKOV3	RNA-Seq	goBP	GO:0001655~urogenital system development	1.87E-09	75	319
SKOV3	RNA-Seq	goBP	GO:0033002~muscle cell proliferation	2.17E-08	44	155
SKOV3	RNA-Seq	goBP	GO:0072001~renal system development	2.33E-08	66	282
SKOV3	RNA-Seq	goBP	GO:0001822~kidney development	3.16E-08	63	266
SKOV3	RNA-Seq	goBP	GO:0032963~collagen metabolic process	3.55E-08	35	110
SKOV3	RNA-Seq	goBP	GO:0044236~multicellular organism metabolic process	6.03E-08	39	133
SKOV3	RNA-Seq	goBP	GO:0044259~multicellular organismal macromolecule metabolic process	1.19E-07	35	115
SKOV3	RNA-Seq	goBP	GO:0050673~epithelial cell proliferation	1.76E-07	74	347
SKOV3	RNA-Seq	goBP	GO:0055123~digestive system development	2.37E-07	40	145
SKOV3	RNA-Seq	goBP	GO:0030335~positive regulation of cell migration	4.02E-07	80	393
SKOV3	RNA-Seq	kegg	hsa05332:Graft-versus-host disease	1.19E-05	14	30
SKOV3	RNA-Seq	kegg	hsa05200:Pathways in cancer	1.28E-05	75	393
SKOV3	RNA-Seq	kegg	hsa05146:Amoebiasis	0.000125	27	105
SKOV3	RNA-Seq	kegg	hsa05219:Bladder cancer	0.00013	15	41
SKOV3	RNA-Seq	kegg	hsa04940:Type I diabetes mellitus	0.000296	14	39
SKOV3	RNA-Seq	kegg	hsa04514:Cell adhesion molecules (CAMs)	0.000532	31	139
SKOV3	RNA-Seq	kegg	hsa04015:Rap1 signaling pathway	0.001753	40	209
SKOV3	RNA-Seq	kegg	hsa05166:HTLV-I infection	0.001876	46	251
SKOV3	RNA-Seq	kegg	hsa04390:Hippo signaling pathway	0.00194	31	150
SKOV3	RNA-Seq	kegg	hsa04668:TNF signaling pathway	0.002421	24	107
SKOV3	RNA-Seq	kegg	hsa04510:Focal adhesion	0.002469	39	206
SKOV3	RNA-Seq	kegg	hsa05416:Viral myocarditis	0.002819	15	54

SKOV3	RNA-Seq	kegg	hsa05217:Basal cell carcinoma	0.002819	15	54
SKOV3	RNA-Seq	kegg	hsa05205:Proteoglycans in cancer	0.003727	37	197
OVCAR3	RNA-Seq	goBP	GO:0042254~ribosome biogenesis	3.4E-08	71	320
OVCAR3	RNA-Seq	goBP	GO:0006364~rRNA processing	7.84E-07	57	256
OVCAR3	RNA-Seq	goBP	GO:0016072~rRNA metabolic process	8.62E-07	58	263
OVCAR3	RNA-Seq	goBP	GO:0034470~ncRNA processing	2.53E-06	77	397
OVCAR3	RNA-Seq	goBP	GO:0009124~nucleoside monophosphate biosynthetic process	3.17E-06	28	94
OVCAR3	RNA-Seq	goBP	GO:0009156~ribonucleoside monophosphate biosynthetic process	4.55E-06	26	85
OVCAR3	RNA-Seq	goBP	GO:0009168~purine ribonucleoside monophosphate biosynthetic process	1.09E-05	23	73
OVCAR3	RNA-Seq	goBP	GO:0009127~purine nucleoside monophosphate biosynthetic process	1.09E-05	23	73
OVCAR3	RNA-Seq	goBP	GO:0030490~maturation of SSU-rRNA	1.73E-05	17	45
OVCAR3	RNA-Seq	goBP	GO:0000479~endonucleolytic cleavage of tricistronic rRNA transcript (SSU-rRNA, 5.8S rRNA, LSU-rRNA)	2E-05	9	13
OVCAR3	RNA-Seq	goBP	GO:0000478~endonucleolytic cleavage involved in rRNA processing	4.2E-05	9	14
OVCAR3	RNA-Seq	goBP	GO:0046040~IMP metabolic process	4.2E-05	9	14
OVCAR3	RNA-Seq	goBP	GO:0061077~chaperone-mediated protein folding	7.61E-05	17	50
OVCAR3	RNA-Seq	goBP	GO:0032534~regulation of microvillus assembly	9.97E-05	6	6
OVCAR3	RNA-Seq	goBP	GO:0042274~ribosomal small subunit biogenesis	0.000116	19	62
OVCAR3	RNA-Seq	kegg	hsa05323:Rheumatoid arthritis	1.63E-05	25	84
OVCAR3	RNA-Seq	kegg	hsa03008:Ribosome biogenesis in eukaryotes	1.84E-05	23	74
OVCAR3	RNA-Seq	kegg	hsa04142:Lysosome	8.48E-05	30	121
OVCAR3	RNA-Seq	kegg	hsa00330:Arginine and proline metabolism	0.000275	16	49
OVCAR3	RNA-Seq	kegg	hsa00230:Purine metabolism	0.000527	36	172
OVCAR3	RNA-Seq	kegg	hsa01100:Metabolic pathways	0.001831	169	1193
OVCAR3	RNA-Seq	kegg	hsa04360:Axon guidance	0.00242	27	127
OVCAR3	RNA-Seq	kegg	hsa05146:Amoebiasis	0.008001	22	105
OVCAR3	RNA-Seq	kegg	hsa04512:ECM-receptor interaction	0.009525	19	87
OVCAR3	RNA-Seq	kegg	hsa01130:Biosynthesis of antibiotics	0.015654	36	210
OVCAR3	RNA-Seq	kegg	hsa05222:Small cell lung cancer	0.016063	18	85
OVCAR3	RNA-Seq	kegg	hsa05332:Graft-versus-host disease	0.017714	9	30

OVCAR3	RNA-Seq	kegg	hsa05133:Pertussis	0.020408	16	74
OVCAR3	RNA-Seq	kegg	hsa05323:Rheumatoid arthritis	1.63E-05	25	84
OVCAR3	RNA-Seq	kegg	hsa03008:Ribosome biogenesis in eukaryotes	1.84E-05	23	74
OVCAR8	RNA-Seq	goBP	GO:0001763~morphogenesis of a branching structure	1.86E-09	28	195
OVCAR8	RNA-Seq	goBP	GO:0061138~morphogenesis of a branching epithelium	4.92E-09	26	177
OVCAR8	RNA-Seq	goBP	GO:0030198~extracellular matrix organization	4.55E-08	35	333
OVCAR8	RNA-Seq	goBP	GO:0043062~extracellular structure organization	4.9E-08	35	334
OVCAR8	RNA-Seq	goBP	GO:0048754~branching morphogenesis of an epithelial tube	6.85E-08	22	147
OVCAR8	RNA-Seq	goBP	GO:0060326~cell chemotaxis	2.89E-07	28	247
OVCAR8	RNA-Seq	goBP	GO:0002040~sprouting angiogenesis	1.07E-06	14	70
OVCAR8	RNA-Seq	goBP	GO:0050673~epithelial cell proliferation	1.12E-06	33	347
OVCAR8	RNA-Seq	goBP	GO:0072001~renal system development	1.24E-06	29	282
OVCAR8	RNA-Seq	goBP	GO:0001655~urogenital system development	1.63E-06	31	319
OVCAR8	RNA-Seq	goBP	GO:0060562~epithelial tube morphogenesis	2.53E-06	30	309
OVCAR8	RNA-Seq	goBP	GO:1901342~regulation of vasculature development	2.62E-06	26	244
OVCAR8	RNA-Seq	goBP	GO:0035239~tube morphogenesis	3.41E-06	32	348
OVCAR8	RNA-Seq	goBP	GO:1904018~positive regulation of vasculature development	3.83E-06	19	143
OVCAR8	RNA-Seq	goBP	GO:0001822~kidney development	3.97E-06	27	266
OVCAR8	RNA-Seq	kegg	hsa04151:PI3K-Akt signaling pathway	0.000304	26	345
OVCAR8	RNA-Seq	kegg	hsa05202:Transcriptional misregulation in cancer	0.000556	16	166
OVCAR8	RNA-Seq	kegg	hsa05323:Rheumatoid arthritis	0.000566	11	84
OVCAR8	RNA-Seq	kegg	hsa05206:MicroRNAs in cancer	0.001073	14	141
OVCAR8	RNA-Seq	kegg	hsa04015:Rap1 signaling pathway	0.002176	17	209
OVCAR8	RNA-Seq	kegg	hsa04621:NOD-like receptor signaling pathway	0.002874	8	56
OVCAR8	RNA-Seq	kegg	hsa04668:TNF signaling pathway	0.003616	11	107
OVCAR8	RNA-Seq	kegg	hsa04930:Type II diabetes mellitus	0.005695	7	48
OVCAR8	RNA-Seq	kegg	hsa04510:Focal adhesion	0.01132	15	206
OVCAR8	RNA-Seq	kegg	hsa05133:Pertussis	0.013254	8	74
OVCAR8	RNA-Seq	kegg	hsa05200:Pathways in cancer	0.015879	23	393
OVCAR8	RNA-Seq	kegg	hsa05321:Inflammatory bowel disease (IBD)	0.017825	7	61
OVCAR8	RNA-Seq	kegg	hsa05332:Graft-versus-host disease	0.018544	5	30

OVCAR8	RNA-Seq	kegg	hsa00410:beta-Alanine metabolism	0.020732	5	31
OVCAR8	RNA-Seq	kegg	hsa05144:Malaria	0.023759	6	48
SKOV3	Proteomic	goBP	GO:0043062~extracellular structure organization	6.83E-09	34	83
SKOV3	Proteomic	goBP	GO:0030198~extracellular matrix organization	6.83E-09	34	83
SKOV3	Proteomic	goBP	GO:0040011~locomotion	7.65E-09	94	374
SKOV3	Proteomic	goBP	GO:0016337~single organismal cell-cell adhesion	2.18E-07	53	182
SKOV3	Proteomic	goBP	GO:0042127~regulation of cell proliferation	1.31E-06	90	391
SKOV3	Proteomic	goBP	GO:0044282~small molecule catabolic process	1.63E-06	40	129
SKOV3	Proteomic	goBP	GO:0009612~response to mechanical stimulus	2.04E-06	22	51
SKOV3	Proteomic	goBP	GO:0098602~single organism cell adhesion	2.56E-06	55	206
SKOV3	Proteomic	goBP	GO:0048870~cell motility	4.76E-06	77	329
SKOV3	Proteomic	goBP	GO:0051674~localization of cell	4.76E-06	77	329
SKOV3	Proteomic	goBP	GO:0003008~system process	4.86E-06	69	285
SKOV3	Proteomic	goBP	GO:0016477~cell migration	4.88E-06	73	307
SKOV3	Proteomic	goBP	GO:0006954~inflammatory response	6.49E-06	34	107
SKOV3	Proteomic	goBP	GO:0044712~single-organism catabolic process	1.26E-05	68	287
SKOV3	Proteomic	goBP	GO:0051270~regulation of cellular component movement	1.65E-05	54	213
SKOV3	Proteomic	kegg	hsa04512:ECM-receptor interaction	0.0001	14	29
SKOV3	Proteomic	kegg	hsa04514:Cell adhesion molecules (CAMs)	0.000103	12	22
SKOV3	Proteomic	kegg	hsa00980:Metabolism of xenobiotics by cytochrome P450	0.000145	9	13
SKOV3	Proteomic	kegg	hsa05204:Chemical carcinogenesis	0.000618	8	12
SKOV3	Proteomic	kegg	hsa01130:Biosynthesis of antibiotics	0.001166	34	132
SKOV3	Proteomic	kegg	hsa04974:Protein digestion and absorption	0.001193	10	20
SKOV3	Proteomic	kegg	hsa00051:Fructose and mannose metabolism	0.002487	9	18
SKOV3	Proteomic	kegg	hsa05205:Proteoglycans in cancer	0.003058	22	77
SKOV3	Proteomic	kegg	hsa01100:Metabolic pathways	0.004472	96	508
SKOV3	Proteomic	kegg	hsa00010:Glycolysis / Gluconeogenesis	0.004635	12	32
SKOV3	Proteomic	kegg	hsa00250:Alanine, aspartate and glutamate metabolism	0.005198	8	16
SKOV3	Proteomic	kegg	hsa00140:Steroid hormone biosynthesis	0.005661	5	6
SKOV3	Proteomic	kegg	hsa05144:Malaria	0.005661	5	6
SKOV3	Proteomic	kegg	hsa00520:Amino sugar and nucleotide sugar metabolism	0.006702	11	29

SKOV3	Proteomic	kegg	hsa01230:Biosynthesis of amino acids	0.009565	15	49

Table S4. DAVID Heatmap positive regulation of cell migration

cellLine	geneset	Gene	Bru.Seq	RNA.Seq	Proteomics
SKOV3	GO:0030335~positive regulation of cell migration	COL1A1	3.10332	8.984349	0
SKOV3	GO:0030335~positive regulation of cell migration	CCL26	2.481395	7.597501	0
SKOV3	GO:0030335~positive regulation of cell migration	SPARC	3.673831	2.390179	2.26
SKOV3	GO:0030335~positive regulation of cell migration	GLIPR2	2.325793	3.134353	2.38
SKOV3	GO:0030335~positive regulation of cell migration	RARRES2	2.360726	5.464708	0
SKOV3	GO:0030335~positive regulation of cell migration	RET	0.611394	7.005418	0
SKOV3	GO:0030335~positive regulation of cell migration	CXCL16	2.301841	5.250767	0
SKOV3	GO:0030335~positive regulation of cell migration	PDGFRB	1.498231	5.819091	0
SKOV3	GO:0030335~positive regulation of cell migration	DAPK2	2.074963	4.9514	0
SKOV3	GO:0030335~positive regulation of cell migration	FLT4	0.301845	6.603341	0
SKOV3	GO:0030335~positive regulation of cell migration	PDGFB	4.562359	2.093152	0
SKOV3	GO:0030335~positive regulation of cell migration	COL18A1	0.454158	3.390885	2.39
SKOV3	GO:0030335~positive regulation of cell migration	FAM83H	0.945706	4.970135	0
SKOV3	GO:0030335~positive regulation of cell migration	DMTN	1.387378	4.357801	0
SKOV3	GO:0030335~positive regulation of cell migration	VEGFA	2.999873	2.665258	0
SKOV3	GO:0030335~positive regulation of cell migration	IL1R1	2.586965	3.051719	0
SKOV3	GO:0030335~positive regulation of cell migration	CTSH	0.361475	5.051075	0.09
SKOV3	GO:0030335~positive regulation of cell migration	ITGB3	1.791232	1.677402	1.7
SKOV3	GO:0030335~positive regulation of cell migration	ADGRA2	1.425032	3.480121	0
SKOV3	GO:0030335~positive regulation of cell migration	EPB41L5	0.681298	2.155933	2.04
SKOV3	GO:0030335~positive regulation of cell migration	BMP4	0.555874	4.23315	0
SKOV3	GO:0030335~positive regulation of cell migration	CXCL14	0	4.639326	0
SKOV3	GO:0030335~positive regulation of cell migration	F3	-1.44182	3.193444	2.72
SKOV3	GO:0030335~positive regulation of cell migration	F2RL1	-0.57772	5.029009	0
SKOV3	GO:0030335~positive regulation of cell migration	GPLD1	-0.01906	3.945277	0
SKOV3	GO:0030335~positive regulation of cell migration	HSPB1	-0.80918	1.433369	3.11
SKOV3	GO:0030335~positive regulation of cell migration	INSR	0.888935	2.79086	0
SKOV3	GO:0030335~positive regulation of cell migration	SHTN1	-0.17351	1.587627	2.18

SKOV3	GO:0030335~positive regulation of cell migration	SNAI1	1.261204	2.331026	0
SKOV3	GO:0030335~positive regulation of cell migration	S1PR1	5.288742	-1.74826	0
SKOV3	GO:0030335~positive regulation of cell migration	CARMIL1	-0.03135	2.047333	1.33
SKOV3	GO:0030335~positive regulation of cell migration	THBS1	-0.73961	1.864538	2
SKOV3	GO:0030335~positive regulation of cell migration	LGALS3	0.734311	0	2.38
SKOV3	GO:0030335~positive regulation of cell migration	TGFB2	3.031233	0	0
SKOV3	GO:0030335~positive regulation of cell migration	NUMB	0.449714	1.253205	1.31
SKOV3	GO:0030335~positive regulation of cell migration	CDH13	-1.52294	4.492922	0
SKOV3	GO:0030335~positive regulation of cell migration	SEMA3F	0.563688	2.374543	0
SKOV3	GO:0030335~positive regulation of cell migration	RHOD	0.217665	2.669986	0
SKOV3	GO:0030335~positive regulation of cell migration	ANXA3	0.231059	1.149312	1.5
SKOV3	GO:0030335~positive regulation of cell migration	MYADM	0.67398	0.765556	1.32
SKOV3	GO:0030335~positive regulation of cell migration	FBLN1	-0.06047	2.585063	0
SKOV3	GO:0030335~positive regulation of cell migration	F2R	-0.04108	2.544282	0
SKOV3	GO:0030335~positive regulation of cell migration	HIF1A	0.44422	1.998386	0
SKOV3	GO:0030335~positive regulation of cell migration	DAB2IP	0.663462	1.565199	0.13
SKOV3	GO:0030335~positive regulation of cell migration	CREB3	0.668535	1.642499	0
SKOV3	GO:0030335~positive regulation of cell migration	TIRAP	0.220221	2.064385	0
SKOV3	GO:0030335~positive regulation of cell migration	ITGA4	-1.93857	4.183326	0
SKOV3	GO:0030335~positive regulation of cell migration	IRS2	2.078247	0.14185	0
SKOV3	GO:0030335~positive regulation of cell migration	EPB41L4B	-0.00081	2.049256	0
SKOV3	GO:0030335~positive regulation of cell migration	FGF2	-0.1607	0.611612	1.49
SKOV3	GO:0030335~positive regulation of cell migration	RHOB	-1.44594	1.3926	1.98
SKOV3	GO:0030335~positive regulation of cell migration	EDN1	-1.44591	3.349144	0
SKOV3	GO:0030335~positive regulation of cell migration	CAMK1D	-0.63734	1.135947	1.37
SKOV3	GO:0030335~positive regulation of cell migration	CSF1	1.231014	0.486557	0
SKOV3	GO:0030335~positive regulation of cell migration	ITGAV	-0.72289	1.057981	1.25
SKOV3	GO:0030335~positive regulation of cell migration	LPAR1	-0.39706	1.866002	0
SKOV3	GO:0030335~positive regulation of cell migration	SRC	1.205133	-0.18792	0.36
SKOV3	GO:0030335~positive regulation of cell migration	KITLG	-0.75743	1.898984	0
SKOV3	GO:0030335~positive regulation of cell migration	HSPA5	1.513889	-0.32613	-0.18

SKOV3	GO:0030335~positive regulation of cell migration	MIEN1	-0.79407	0.603503	1.09
SKOV3	GO:0030335~positive regulation of cell migration	SYNE2	-0.74446	0.444829	1.1
SKOV3	GO:0030335~positive regulation of cell migration	SERPINE1	1.081917	-1.11384	0.65
SKOV3	GO:0030335~positive regulation of cell migration	KIT	-2.32386	2.641013	0
SKOV3	GO:0030335~positive regulation of cell migration	SEMA3A	-1.33607	1.193183	0
SKOV3	GO:0030335~positive regulation of cell migration	IL12A	1.706235	-2.06575	0
SKOV3	GO:0030335~positive regulation of cell migration	ARHGEF39	-1.72755	1.189808	0
SKOV3	GO:0030335~positive regulation of cell migration	ONECUT2	-1.14046	0.595967	0
SKOV3	GO:0030335~positive regulation of cell migration	CALR	-1.0244	-0.11158	0.49
SKOV3	GO:0030335~positive regulation of cell migration	CXCL2	1.472821	-2.25331	0
SKOV3	GO:0030335~positive regulation of cell migration	DDR2	1.809679	-2.60867	0
SKOV3	GO:0030335~positive regulation of cell migration	CIB1	-1.04941	0	0
SKOV3	GO:0030335~positive regulation of cell migration	NRP2	-1.09832	-0.00779	-0.07
SKOV3	GO:0030335~positive regulation of cell migration	SOX9	2.143143	-3.40061	0
SKOV3	GO:0030335~positive regulation of cell migration	NRP1	-1.30644	0	0
SKOV3	GO:0030335~positive regulation of cell migration	TNFRSF14	3.481329	-4.82813	0
SKOV3	GO:0030335~positive regulation of cell migration	SEMA3C	-1.12498	-0.25574	0
SKOV3	GO:0030335~positive regulation of cell migration	GTSE1	-1.36663	-0.0882	0
SKOV3	GO:0030335~positive regulation of cell migration	SDCBP	0.338721	-0.63351	-1.19
SKOV3	GO:0030335~positive regulation of cell migration	SEMA3E	0.758704	-2.30006	0
SKOV3	GO:0030335~positive regulation of cell migration	CORO1A	-3.98865	2.213138	0
SKOV3	GO:0030335~positive regulation of cell migration	PIK3R1	-1.94238	0.116306	0
SKOV3	GO:0030335~positive regulation of cell migration	SNAI2	-2.13353	0.049529	0
SKOV3	GO:0030335~positive regulation of cell migration	CCBE1	-2.10284	-0.07531	0
SKOV3	GO:0030335~positive regulation of cell migration	PIK3CD	-0.2409	-1.94828	0
SKOV3	GO:0030335~positive regulation of cell migration	TGFBR2	-0.3302	-1.93938	0
SKOV3	GO:0030335~positive regulation of cell migration	PLAU	-0.28708	-2.00841	0
SKOV3	GO:0030335~positive regulation of cell migration	SEMA4B	-1.94822	-0.3625	0
SKOV3	GO:0030335~positive regulation of cell migration	PDGFC	-1.34176	-0.98826	0
SKOV3	GO:0030335~positive regulation of cell migration	PRKCE	-0.07468	-2.30385	0
SKOV3	GO:0030335~positive regulation of cell migration	LAMB1	-2.00817	-0.31351	-0.07

SKOV3	GO:0030335~positive regulation of cell migration	GATA3	5.066343	-7.45829	0
SKOV3	GO:0030335~positive regulation of cell migration	CXCL3	0.35076	-2.87211	0
SKOV3	GO:0030335~positive regulation of cell migration	CYR61	0.574282	-2.04739	-1.1
SKOV3	GO:0030335~positive regulation of cell migration	MDM2	-0.15197	-2.65669	0
SKOV3	GO:0030335~positive regulation of cell migration	GPSM3	-0.32376	-2.49201	0
SKOV3	GO:0030335~positive regulation of cell migration	ADAM8	0.524233	-3.66782	0
SKOV3	GO:0030335~positive regulation of cell migration	ITGA5	-0.41871	-1.60538	-1.2
SKOV3	GO:0030335~positive regulation of cell migration	NTF3	5.651321	-8.92975	0
SKOV3	GO:0030335~positive regulation of cell migration	MMP9	1.038781	-4.3604	0
SKOV3	GO:0030335~positive regulation of cell migration	ITGA6	-0.83299	-1.90666	-0.82
SKOV3	GO:0030335~positive regulation of cell migration	SASH1	-0.63608	-1.91574	-1.08
SKOV3	GO:0030335~positive regulation of cell migration	KIF20B	-1.33539	-1.91667	-0.45
SKOV3	GO:0030335~positive regulation of cell migration	IL1A	2.689469	-6.48	0
SKOV3	GO:0030335~positive regulation of cell migration	WNT7A	-4.29391	-0.22309	0
SKOV3	GO:0030335~positive regulation of cell migration	HDAC9	-0.91171	-3.77059	0
SKOV3	GO:0030335~positive regulation of cell migration	SMURF2	-0.6323	-2.51026	-1.71
SKOV3	GO:0030335~positive regulation of cell migration	CXCL1	-1.36623	0	-3.69
SKOV3	GO:0030335~positive regulation of cell migration	ETS1	-1.0379	-2.36536	-1.76
SKOV3	GO:0030335~positive regulation of cell migration	LEF1	0.913275	-6.37594	0
SKOV3	GO:0030335~positive regulation of cell migration	LAMC2	-1.6271	-2.47663	-1.59
SKOV3	GO:0030335~positive regulation of cell migration	TIAM1	-4.08036	-2.03095	0
SKOV3	GO:0030335~positive regulation of cell migration	PGF	-1.76853	-4.39346	0
SKOV3	GO:0030335~positive regulation of cell migration	STAT5A	-2.42061	-3.86168	0
SKOV3	GO:0030335~positive regulation of cell migration	RAC2	1.038781	-7.0022	-0.86
SKOV3	GO:0030335~positive regulation of cell migration	SPHK1	0.03881	-4.7029	-2.49
SKOV3	GO:0030335~positive regulation of cell migration	FOXF1	0	-7.88095	0
SKOV3	GO:0030335~positive regulation of cell migration	SRPX2	-2.35349	-5.54315	0
SKOV3	GO:0030335~positive regulation of cell migration	WNT5B	0.44217	-8.53455	0
SKOV3	GO:0030335~positive regulation of cell migration	DOCK4	-2.59911	-5.57192	0
SKOV3	GO:0030335~positive regulation of cell migration	FGF1	-4.28309	-3.95906	0
SKOV3	GO:0030335~positive regulation of cell migration	ANGPT1	-3.5055	-6.08306	0

SKOV3	GO:0030335~positive regulation of cell migration	MMP14	-0.22422	-8.65711	-2.29
SKOV3	GO:0030335~positive regulation of cell migration	PAK3	-6.58901	-6.09617	0
SKOV3	GO:0030335~positive regulation of cell migration	NOX4	-6.10071	-9.93043	0

Table S5. GSEA analysis Top 10 Gene sets positively/ negatively correlated with *STAT3* KO/WT

cellLine	dataType	geneSet	NES	pValue	qValue	Total GeneSet Genes
SKOV3	Bru-Seq	HALLMARK_INTERFERON_GAMMA_RESPONSE	2.516667	0	0	72
SKOV3	Bru-Seq	HALLMARK_INTERFERON_ALPHA_RESPONSE	2.438759	0	0	40
SKOV3	Bru-Seq	HALLMARK_ALLOGRAFT_REJECTION	2.034693	0	0.000467	62
SKOV3	Bru-Seq	HALLMARK_UNFOLDED_PROTEIN_RESPONSE	2.027282	0	0.000395	83
SKOV3	Bru-Seq	HALLMARK_P53_PATHWAY	1.883627	0	0.001987	105
SKOV3	Bru-Seq	HALLMARK_INFLAMMATORY_RESPONSE	1.632981	0.006297	0.029573	67
SKOV3	Bru-Seq	HALLMARK_TNFA_SIGNALING_VIA_NFKB	1.584272	0.002102	0.037639	111
SKOV3	Bru-Seq	HALLMARK_MTORC1_SIGNALING	1.575598	0.001161	0.035116	151
SKOV3	Bru-Seq	HALLMARK_HYPOXIA	1.542655	0.005624	0.040988	97
SKOV3	Bru-Seq	HALLMARK_IL6_JAK_STAT3_SIGNALING	1.491357	0.03932	0.056163	34
SKOV3	Bru-Seq	HALLMARK_E2F_TARGETS	-2.56518	0	0	177
SKOV3	Bru-Seq	HALLMARK_G2M_CHECKPOINT	-2.52893	0	0	183
SKOV3	Bru-Seq	HALLMARK_MITOTIC_SPINDLE	-1.99353	0	0.000189	158
SKOV3	Bru-Seq	HALLMARK_EPITHELIAL_MESENCHYMAL_TRANSITION	-1.76499	0.000444	0.00711	77
SKOV3	Bru-Seq	HALLMARK_SPERMATOGENESIS	-1.60614	0.009293	0.036225	45
SKOV3	Bru-Seq	HALLMARK_ANGIOGENESIS	-1.41681	0.087822	0.171151	15
SKOV3	Bru-Seq	HALLMARK_MYOGENESIS	-1.38923	0.071631	0.182506	42
SKOV3	Bru-Seq	HALLMARK_UV_RESPONSE_DN	-1.24155	0.122525	0.437976	98
SKOV3	Bru-Seq	HALLMARK_MYC_TARGETS_V1	-1.22605	0.1011	0.427541	186
SKOV3	Bru-Seq	HALLMARK_APICAL_JUNCTION	-1.15249	0.229619	0.578581	71
SKOV3	RNA-Seq	HALLMARK_INTERFERON_ALPHA_RESPONSE	2.299748	0	0	89
SKOV3	RNA-Seq	HALLMARK_INTERFERON_GAMMA_RESPONSE	2.012223	0	0	159
SKOV3	RNA-Seq	HALLMARK_KRAS_SIGNALING_DN	1.803419	0.000153	0.000967	195
SKOV3	RNA-Seq	HALLMARK_MYOGENESIS	1.447885	0.012665	0.097872	195
SKOV3	RNA-Seq	HALLMARK_ESTROGEN_RESPONSE_LATE	1.428453	0.010627	0.095324	192
SKOV3	RNA-Seq	HALLMARK_NOTCH_SIGNALING	1.415264	0.065901	0.090623	72
SKOV3	RNA-Seq	HALLMARK_ANGIOGENESIS	1.408743	0.075971	0.082633	57

SKOV3	RNA-Seq	HALLMARK_COAGULATION	1.389052	0.039242	0.087899	194
SKOV3	RNA-Seq	HALLMARK_ESTROGEN_RESPONSE_EARLY	1.251318	0.072233	0.252189	126
SKOV3	RNA-Seq	HALLMARK_UV_RESPONSE_DN	1.14947	0.19754	0.458058	170
SKOV3	RNA-Seq	HALLMARK_MYC_TARGETS_V1	-2.11507	0	0.000156	89
SKOV3	RNA-Seq	HALLMARK_MYC_TARGETS_V2	-2.0635	0.000279	0.00016	159
SKOV3	RNA-Seq	HALLMARK_E2F_TARGETS	-1.84823	0	0.002048	195
SKOV3	RNA-Seq	HALLMARK_G2M_CHECKPOINT	-1.74371	0	0.00728	195
SKOV3	RNA-Seq	HALLMARK_KRAS_SIGNALING_UP	-1.6461	0.00069	0.018228	192
SKOV3	RNA-Seq	HALLMARK_TNFA_SIGNALING_VIA_NFKB	-1.5468	0.00076	0.03995	72
SKOV3	RNA-Seq	HALLMARK_OXIDATIVE_PHOSPHORYLATION	-1.54312	0.000388	0.035481	57
SKOV3	RNA-Seq	HALLMARK_REACTIVE_OXIGEN_SPECIES_PATHWAY	-1.53436	0.01762	0.03311	194
SKOV3	RNA-Seq	HALLMARK_WNT_BETA_CATENIN_SIGNALING	-1.42839	0.056524	0.074144	126
SKOV3	RNA-Seq	HALLMARK_EPITHELIAL_MESENCHYMAL_TRANSITION	-1.3677	0.018015	0.108846	170
OVCAR3	RNA-Seq	HALLMARK_APOPTOSIS	1.992997	0	0.000407	128
OVCAR3	RNA-Seq	HALLMARK_EPITHELIAL_MESENCHYMAL_TRANSITION	1.960474	0	0.000505	127
OVCAR3	RNA-Seq	HALLMARK_INFLAMMATORY_RESPONSE	1.900985	0	0.001246	105
OVCAR3	RNA-Seq	HALLMARK_INTERFERON_ALPHA_RESPONSE	1.866575	0.000262	0.001572	77
OVCAR3	RNA-Seq	HALLMARK_COMPLEMENT	1.845507	0	0.00173	123
OVCAR3	RNA-Seq	HALLMARK_KRAS_SIGNALING_UP	1.843282	0.000125	0.001477	105
OVCAR3	RNA-Seq	HALLMARK_TNFA_SIGNALING_VIA_NFKB	1.8329	0	0.001463	167
OVCAR3	RNA-Seq	HALLMARK_XENOBIOTIC_METABOLISM	1.822422	0	0.001435	131
OVCAR3	RNA-Seq	HALLMARK_INTERFERON_GAMMA_RESPONSE	1.819007	0.000119	0.00132	145
OVCAR3	RNA-Seq	HALLMARK_COAGULATION	1.791311	0.000401	0.001646	69
OVCAR3	RNA-Seq	HALLMARK_MYC_TARGETS_V1	-2.99828	0	0	128
OVCAR3	RNA-Seq	HALLMARK_MYC_TARGETS_V2	-2.80263	0	0	127
OVCAR3	RNA-Seq	HALLMARK_E2F_TARGETS	-2.10334	0	0.000509	105
OVCAR3	RNA-Seq	HALLMARK_G2M_CHECKPOINT	-1.72499	0	0.01031	77
OVCAR3	RNA-Seq	HALLMARK_UNFOLDED_PROTEIN_RESPONSE	-1.23426	0.098435	0.236274	123
OVCAR3	RNA-Seq	HALLMARK_MTORC1_SIGNALING	-1.20083	0.088079	0.241515	105
OVCAR3	RNA-Seq	HALLMARK_ANDROGEN_RESPONSE	-1.04776	0.345618	0.49203	167
OVCAR3	RNA-Seq	HALLMARK_DNA_REPAIR	-0.93212	0.63376	0.750694	131

OVCAR3	RNA-Seq	HALLMARK_OXIDATIVE_PHOSPHORYLATION	-0.89638	0.78439	0.758273	145
OVCAR3	RNA-Seq	HALLMARK_TGF_BETA_SIGNALING	-0.77035	0.859516	0.905734	69
OVCAR8	RNA-Seq	HALLMARK_MYC_TARGETS_V2	1.8636	0.000256	0.006548	58
OVCAR8	RNA-Seq	HALLMARK_FATTY_ACID_METABOLISM	1.649553	0.000312	0.043075	121
OVCAR8	RNA-Seq	HALLMARK_OXIDATIVE_PHOSPHORYLATION	1.474128	0.002943	0.136384	194
OVCAR8	RNA-Seq	HALLMARK_GLYCOLYSIS	1.353332	0.01767	0.2554	172
OVCAR8	RNA-Seq	HALLMARK_ESTROGEN_RESPONSE_EARLY	1.211118	0.101604	0.539366	148
OVCAR8	RNA-Seq	HALLMARK_HEME_METABOLISM	1.204223	0.101151	0.470015	157
OVCAR8	RNA-Seq	HALLMARK_MTORC1_SIGNALING	1.160292	0.127532	0.528075	191
OVCAR8	RNA-Seq	HALLMARK_XENOBIOTIC_METABOLISM	1.121209	0.202071	0.585024	130
OVCAR8	RNA-Seq	HALLMARK_P53_PATHWAY	1.066742	0.279308	0.704982	171
OVCAR8	RNA-Seq	HALLMARK_MYC_TARGETS_V1	1.057282	0.299628	0.667088	195
OVCAR8	RNA-Seq	HALLMARK_TNFA_SIGNALING_VIA_NFKB	-2.02545	0	0.001931	58
OVCAR8	RNA-Seq	HALLMARK_KRAS_SIGNALING_UP	-1.8768	0	0.010079	121
OVCAR8	RNA-Seq	HALLMARK_INFLAMMATORY_RESPONSE	-1.80619	0.000751	0.015834	194
OVCAR8	RNA-Seq	HALLMARK_ALLOGRAFT_REJECTION	-1.64194	0.006975	0.060156	172
OVCAR8	RNA-Seq	HALLMARK_EPITHELIAL_MESENCHYMAL_TRANSITION	-1.60691	0.006083	0.066272	148
OVCAR8	RNA-Seq	HALLMARK_MYOGENESIS	-1.58272	0.01029	0.067778	157
OVCAR8	RNA-Seq	HALLMARK_COAGULATION	-1.52259	0.031406	0.095049	191
OVCAR8	RNA-Seq	HALLMARK_IL2_STAT5_SIGNALING	-1.51455	0.013374	0.08831	130
OVCAR8	RNA-Seq	HALLMARK_IL6_JAK_STAT3_SIGNALING	-1.50008	0.043156	0.087327	171
OVCAR8	RNA-Seq	HALLMARK_PANCREAS_BETA_CELLS	-1.48937	0.071815	0.085294	195
SKOV3	Proteomic	HALLMARK_MYOGENESIS	1.879868	0.000179	0.007364	54
SKOV3	Proteomic	HALLMARK_UV_RESPONSE_DN	1.706487	0.003383	0.033606	55
SKOV3	Proteomic	HALLMARK_APICAL_JUNCTION	1.687344	0.001593	0.027547	71
SKOV3	Proteomic	HALLMARK_XENOBIOTIC_METABOLISM	1.642826	0.003581	0.032667	69
SKOV3	Proteomic	HALLMARK_ESTROGEN_RESPONSE_LATE	1.613413	0.005527	0.036539	64
SKOV3	Proteomic	HALLMARK_HYPOXIA	1.546624	0.011739	0.058273	70
SKOV3	Proteomic	HALLMARK_INTERFERON_GAMMA_RESPONSE	1.472083	0.028525	0.09672	63
SKOV3	Proteomic	HALLMARK_P53_PATHWAY	1.470959	0.024862	0.085254	71
SKOV3	Proteomic	HALLMARK_INTERFERON_ALPHA_RESPONSE	1.430651	0.05216	0.104046	33

SKOV3	Proteomic	HALLMARK_GLYCOLYSIS	1.325278	0.06177	0.204928	96
SKOV3	Proteomic	HALLMARK_MYC_TARGETS_V2	-1.97122	0	0.002563	51
SKOV3	Proteomic	HALLMARK_G2M_CHECKPOINT	-1.81457	0	0.010523	131
SKOV3	Proteomic	HALLMARK_MYC_TARGETS_V1	-1.79936	0	0.008355	188
SKOV3	Proteomic	HALLMARK_KRAS_SIGNALING_UP	-1.71193	0.011189	0.016199	26
SKOV3	Proteomic	HALLMARK_E2F_TARGETS	-1.61005	0.000489	0.034282	149
SKOV3	Proteomic	HALLMARK_TNFA_SIGNALING_VIA_NFKB	-1.56307	0.012027	0.042148	49
SKOV3	Proteomic	HALLMARK_IL6_JAK_STAT3_SIGNALING	-1.54057	0.041277	0.043386	17
SKOV3	Proteomic	HALLMARK_EPITHELIAL_MESENCHYMAL_TRANSITION	-1.47822	0.0271	0.062809	66
SKOV3	Proteomic	HALLMARK_DNA_REPAIR	-1.35037	0.045315	0.138883	95
SKOV3	Proteomic	HALLMARK_ESTROGEN_RESPONSE_EARLY	-1.32248	0.086207	0.150363	53

Table S6. 39 differentially expressed genes identified by multi-omic analyses in response to *STAT3* KO

**Common genes in SKOV3 Multi-omic**		**Common genes across 3 cell lines**	
**Up-regulated**	**Down-regulated**	**Up-regulated**	**Down-regulated**
ASS1	CXCL1	ANGPTL4	ALDH1A3
COL5A1	ETS1	CD74	GLIPR1
CTH	FABP5	CPA4	KRTAP2-3
EPB41L1	FOSL1	HOGA1	PRSS12
NXN	HMGA1	IFIT2	S100A1
SPANXB1	HMGA2	IFIT3	STMN3
STAT1	LAMB3	L1CAM	
TAP1	LAMC2	NEURL1	
ASS1	MARCKS	PRR5L	
	MCM2	PSG4	
	NT5E	RGL3	
	PLEK2	SERINC2	
	PSMD2	SH2D3A	
	STARD13		

Table S7. 14 epithelial- or mesenchymal-related annotated gene sets adopted from the Molecular Signatures Database (MSigDB, GSEA - Broad Institute)

Gene sets	# genes	Discription
EPITHELIAL_TO_MESENCHYMAL_TRANSITION	10	14 epithelial- or mesenchymal-related annotated gene sets are adopted from the Molecular Signatures Database (MSigDB, GSEA - Broad Institute).
GO_CARDIAC_EPITHELIAL_TO_MESENCHYMAL_TRANSITION	24	A transition where a cardiac epithelial cell loses apical/basolateral polarity, severs intercellular adhesive junctions, degrades basement membrane components and becomes a migratory mesenchymal cell.
GO_EPITHELIAL_TO_MESENCHYMAL_TRANSITION	56	A transition where an epithelial cell loses apical/basolateral polarity, severs intercellular adhesive junctions, degrades basement membrane components and becomes a migratory mesenchymal cell.
GO_EPITHELIAL_TO_MESENCHYMAL_TRANSITION_INVOLVED_IN_ENDOCARDIAL_CUSHION_FORMATION	11	A transition where a cardiac epithelial cell loses apical/basolateral polarity, severs intercellular adhesive junctions, degrades basement membrane components and becomes a migratory mesenchymal cell that will contribute to the formation of the endocardial cushion.
GO_MESENCHYMAL_TO_EPITHELIAL_TRANSITION	15	A transition where a mesenchymal cell establishes apical/basolateral polarity, forms intercellular adhesive junctions, synthesizes basement membrane components and becomes an epithelial cell.
GO_NEGATIVE_REGULATION_OF_EPITHELIAL_TO_MESENCHYMAL_TRANSITION	23	Any process that decreases the rate, frequency, or extent of epithelial to mesenchymal transition. Epithelial to mesenchymal transition where an epithelial cell loses apical/basolateral polarity, severs intercellular adhesive junctions, degrades basement membrane components and becomes a migratory mesenchymal cell.
GO_POSITIVE_REGULATION_OF_EPITHELIAL_TO_MESENCHYMAL_TRANSITION	34	Any process that increases the rate, frequency, or extent of epithelial to mesenchymal transition. Epithelial to mesenchymal transition is where an epithelial cell loses apical/basolateral polarity, severs intercellular adhesive junctions, degrades basement membrane components and becomes a migratory mesenchymal cell.

GOTZMANN_EPITHELIAL_TO_MESENCHYMAL_TRANSITION_DN	206	Genes down-regulated in MMH-RT cells (hepatocytes displaying an invasive, metastatic phenotype) during epithelial to mesenchymal transition (EMT).
GOTZMANN_EPITHELIAL_TO_MESENCHYMAL_TRANSITION_UP	69	Genes up-regulated in MMH-RT cells (hepatocytes displaying an invasive, metastatic phenotype) during epithelial to mesenchymal transition (EMT).
HALLMARK_EPITHELIAL_MESENCHYMAL_TRANSITION	200	Genes defining epithelial-mesenchymal transition, as in wound healing, fibrosis and metastasis.
JECHLINGER_EPITHELIAL_TO_MESENCHYMAL_TRANSITION_DN	66	Genes down-regulated during epithelial to mesenchymal transition (EMT) induced by TGFB1 [GeneID=7040] in the EpH4 cells (mammary epithelium cell line transformed by HRAS [GeneID=3265]).
JECHLINGER_EPITHELIAL_TO_MESENCHYMAL_TRANSITION_UP	71	Genes up-regulated during epithelial to mesenchymal transition (EMT) induced by TGFB1 [GeneID=7040] in the EpH4 cells (mammary epithelium cell line transformed by HRAS [GeneID=3265]).
SARRIO_EPITHELIAL_MESENCHYMAL_TRANSITION_DN	154	Genes down-regulated in MCF10A cells (breast cancer) grown at low (mesenchymal phenotype) compared to those grown at high (epithelial, basal-like phenotype) confluency.
SARRIO_EPITHELIAL_MESENCHYMAL_TRANSITION_UP	180	Genes up-regulated in MCF10A cells (breast cancer) grown at low (mesenchymal phenotype) compared to those grown at high (epithelial, basal-like phenotype) confluency.

Table S8. Percentage of cell population detected by cell cycle analysis

	SKOV3		OVCAR3		OVCAR8		HEY	
	WT	*STAT3* KO	WT	*STAT3* KO	WT	*STAT3* KO	WT	*STAT3* KO
G1	59.47 ± 4.65	47.73 ± 3.61	53.60 ± 4.06	37.70 ± 3.61	45.28 ± 7.21	42.00 ± 5.83	59.37 ± 2.71	61.97 ± 8.31
S	16.67 ± 1.81	12.13 ± 2.00	16.73 ± 2.14	17.10 ± 2.19	19.43 ± 7.17	17.18 ± 1.61	16.97 ± 0.59	12.93 ± 0.76
G2/M	22.90 ± 5.45	35.87 ± 0.61	28.60 ± 3.66	41.43 ± 6.90	34.53 ± 7.31	39.80 ± 3.81	22.50 ± 2.40	24.00 ± 8.54

All data is represented as mean ± standard error of the mean.

Supplementary Data 1 Pathology report for SKOV3 tumor

Hematoxylin and eosin (H&E)- stained slides of two xenograft tumors (SKOV3 WT and SKOV3STAT3 KO) were submitted for histologic interpretation.

RESULTS (descriptive):

SKOV3 WT: Submitted are two serial histologic sections of two samples of xenograft tumor, each measuring approximately 0.5 X 1.0cm, and stained with hematoxylin and eosin (H&E). Each section was composed of an expansile, locally infiltrative proliferation of solid lobules, clusters, and packets of poorly differentiated epithelial cells within a fine fibrovascular stroma, with local invasion focally into adjacent skeletal muscle, and central necrosis evidenced by hypereosinophilic cellular debris. Tumor cells were polygonal to cuboidal, with scant to moderate faintly eosinophilic amorphous to vacuolated cytoplasm, round to reniform hyperchromatic nuclei with finely clumped marginated chromatin, and up to two discrete nucleoli. There was moderate variation in cellular size and shape (anisocytosis), and nuclear size and shape (anisokaryosis), and there were approximately 0-4 mitoses/high power (40X) field.

SKOVSTAT3 KO: Submitted are four H&E stained serial histologic sections of one xenograft tumor, measuring approximately 0.1 X 0.1cm. In each section, the submitted tissue was composed of similar neoplastic cells, arranged in ribbons and clusters with rare acinar forms, embedded in moderate amounts of collagenous stroma, with local invasion into adjacent skeletal muscle. Tumor cells were polygonal to cuboidal, with scant eosinophilic amorphous cytoplasm and large round to reniform hyperchromatic nuclei with dense to finely clumped marginated chromatin and occasional single nucleoli. There was mild to moderate anisocytosis and anisokaryosis, and 0-3 mitoses/high power (40X) field. There were scattered shrunken and pyknotic cells multifocally within the tumor section.

DISCUSSION:

The purpose of this evaluation was to histologically evaluate the submitted xenograft tumors. There were significant histologic differences between the WT and STAT3 KO tumor specimens. In the STAT3 KO specimen, overall histologic tumor size on the slide was markedly reduced in comparison to the WT specimen, and the cellular density was clearly decreased histologically. In contrast to the WT specimen, which was composed of robust clusters and lobules containing abundant tumor cells within a scant fine fibrovascular stroma, the KO specimen was composed of fewer tumor cells in less distinct lobules, ribbons, and sometimes singly, within a more abundant collagenous stroma. The overall appearance of the KO specimen was that of collapse, with fewer tumor cells and more stroma than that of the WT specimen. Cellular features between the two specimens were also observably different. In the KO specimen, there was relatively less anisocytosis and anisokaryosis, with smaller cells, many of which contained more dense cytoplasm and fewer abnormal nuclear features. Finally, while the WT specimen showed central necrosis characteristic of central tumor hypoxia and ischemic change, the KO specimen showed scattered pyknotic or shrunken cells throughout the specimen, suggesting individual cell degeneration.

Overall, histologic findings in the two submitted specimens significantly differed, with the KO specimen showing decreased cellular density and pleomorphism, increased stroma, and scattered cellular degeneration.
